# Universal NicE-seq for high-resolution accessible chromatin profiling for formaldehyde-fixed and FFPE tissues

**DOI:** 10.1186/s13148-020-00921-6

**Published:** 2020-09-22

**Authors:** Hang Gyeong Chin, Zhiyi Sun, Udayakumar S. Vishnu, Pengying Hao, Paloma Cejas, George Spracklin, Pierre-Olivier Estève, Shuang-yong Xu, Henry W. Long, Sriharsa Pradhan

**Affiliations:** 1 New England Biolabs, Inc., 240 County Road, Ipswich, MA 01938 USA; 2 Center for Functional Cancer Epigenetics, Dana-Farber Cancer Institute, 450 Brookline Avenue, Boston, MA 02215-5450 USA; 3 Translational Oncology Laboratory, Hospital La Paz Institute for Health Research (IdiPAZ) and CIBERONC, La Paz University Hospital, Madrid, Spain

## Abstract

Accessible chromatin plays a central role in gene expression and chromatin architecture. Current accessible chromatin approaches depend on limited digestion/cutting and pasting adaptors at the accessible DNA, thus requiring additional materials and time for optimization. Universal NicE-seq (UniNicE-seq) is an improved accessible chromatin profiling method that negates the optimization step and is suited to a variety of mammalian cells and tissues. Addition of 5-methyldeoxycytidine triphosphate during accessible chromatin labeling and an on-bead library making step substantially improved the signal to noise ratio while protecting the accessible regions from repeated nicking in cell lines, mouse T cells, mouse kidney, and human frozen tissue sections. We also demonstrate one tube UniNicE-seq for the FFPE tissue section for direct NGS library preparation without sonication and DNA purification steps. These refinements allowed reliable mapping of accessible chromatin for high-resolution genomic feature studies.

## Introduction

The eukaryotic nuclear genome is packaged into chromatin, consisting primarily of DNA, proteins, and RNA, which is further condensed into larger folded chromosome structures during cell division. During cellular events, chromatin undergoes remodeling providing accessibility to DNA-binding proteins including transcription factors [1–3]. Gene promoters and enhancers participate in gene expression and confer to accessible chromatin structure. Recent genome-wide methods and studies for mapping chromatin accessibility (open chromatin), nucleosome positioning, and transcription factor occupancy have utilized a variety of methods including DNase hypersensitive region sequencing (DNase-seq) [4], Assay for Transposase-Accessible Chromatin using sequencing (ATAC-seq) [5], and nicking enzyme assisted sequencing (NicE-seq) [6]. Although both DNase-seq and ATAC-seq are powerful methods, they both require specific reagents and cell type-specific optimization including cell number to enzyme/Tn5 transposon concentration and time of incubation [4, 5]. While ATAC-seq works primarily on unfixed cells, mitochondrial DNA sequence contamination was a major issue until a modified Omni-ATAC-seq protocol was developed [7]. Our previously published NicE-Seq method also required similar enzyme titration in each cell type to determine an optimal enzyme to cell ratio for effective labeling and capture of accessible chromatin regions. Therefore, all the current methods required a careful titration of cells to enzyme and incubation time for optimal digestion to capture accessible chromatins. Here, we report an improved, fast, accurate, and robust method, UniNicE-seq, which generates a higher data quality for interrogation of accessible chromatin and eliminating the need for a cell number to enzyme titration. In the refinement to the previous NicE-seq protocol, nuclei are incubated with Nt.CviPII, which nicks human genomic DNA with sequence specificity CCD (*D* = A/G/T) followed by labeling reaction with 5-methyldeoxycytidine triphosphate (5-mdCTP) and biotin-14-dCTP in the dNTPs mixture to specifically label all available deoxycytidine triphosphates at the nicking sites. This will render these sites in the newly labeled accessible chromatin DNA resistant to further nicking. Therefore, even with the excess nicking enzyme, the labeled chromatin is protected from further degradation. After the labeling reaction, biotin-labeled DNA is isolated, sonicated, and captured on streptavidin magnetic beads for library preparation and sequencing. We tested this method on a diverse set of cell lines, native, and formaldehyde-fixed tissue nuclei, fresh frozen formaldehyde-fixed 5–10 μm human tissue sections along with FFPE tissue sections and observed markedly improved accessible chromatin signals. Taken together, universal NicE-seq is a simple, cost-effective, nick translation-based method to profile accessible chromatin using NextGen sequencing. Importantly, 5mC-incorporated accessible chromatin remains resistant to further degradation eliminating time-consuming titration and would allow automation on a variety of biological samples.

## Results

### Universal NicE-seq protects labeled accessible chromatin against enzymatic degradation

**Fig. 1:**
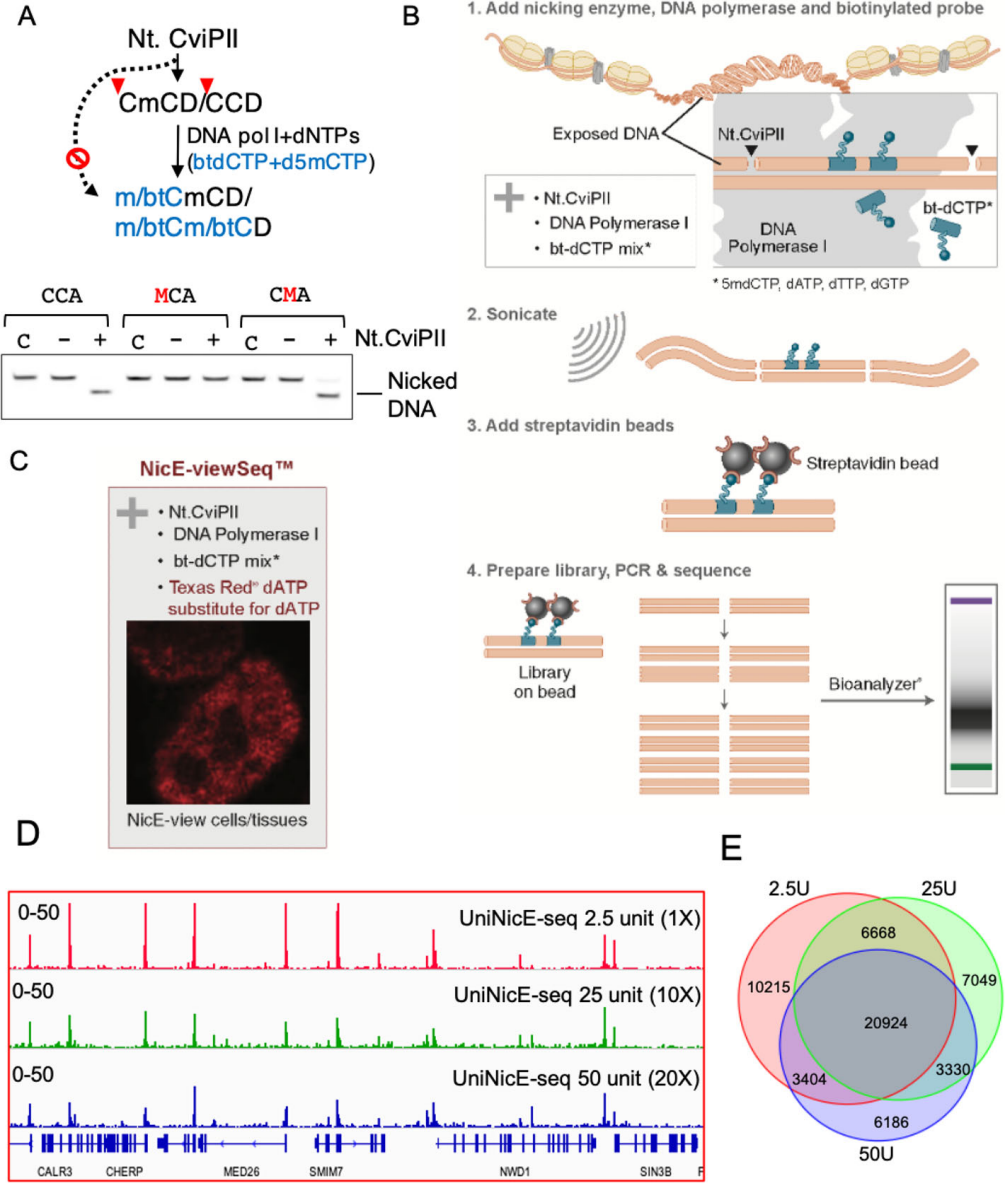
Addition of 5-mdCTP in UniNicE-seq workflow. **a** Schematic diagram showing Nt.CviPII is blocked by 5-methylcytosine in recognition (CCD) sequence. During nick translation, 5-mdCTP can be incorporated at one or both cytosine positions. Nucleotide mixtures containing biotinylated dCTP and 5-mdCTP would allow both labels at CCD sites. Lower panel shows the blocking of nicking by the presence of 5mC at CCD sites. After the nicking reaction, the denatured oligonucleotides were resolved in a urea-acrylamide denaturing gel by electrophoresis. C is control input oligos, − or + indicate the absence or presence of Nt.CviPII in the reaction. mC and btC represent the 5-methylcytosine and biotinylated-cytosine, respectively. Nicked oligonucleotide migrates faster on the denaturing urea gel. **b** Schematic diagram of the UniNicE-seq method for accessible chromatin library preparation. **c** Substitution of dATP by Texas Red-conjugated dATP will allow accessible chromatin visualization in the nucleus. **d** IGV screenshot of the normalized read density of UniNicE-seq using 2.5 U (top track), 25 U (middle track), and 50 U (bottom track) of Nt.CviPII in HCT116 **e** Venn diagram showing the overlap of peaks in various amounts of Nt.CviPII in the universal NicE-seq (UniNicE-seq) reaction in HCT116 cells.

During the previously published NicE-seq labeling reaction, the nucleotide mixture contained dNTPs supplemented with biotin-14-dATP and biotin-14-dCTP [6]. This resulted in labeling accessible chromatin with biotinylated nucleotides for library preparation and enrichment. However, in the presence of an excessive enzyme, the accessible regions were repeatedly nicked resulting in poor library quality and a higher signal to background ratio. In fact, the biochemical property of Nt.CviPII would allow the nicking of an unmodified CCD but not mCCD (Fig. 1a). To prevent repeated nicking at the same site, we substituted the dCTP in the labeling reaction with of 5-methyldeoxycytidine triphosphate (5-mdCTP). Thus, all available deoxycytidine triphosphates were indeed either 5-mdCTP or biotin-14-dCTP. This modification ensured the incorporation of 5-mdCTP and/or biotin-14-dCTP at the nicking sites of Nt.CviPII by DNA Pol I. Modification of CCD sites at the 5’ cytosine renders Nt.CviPII resistant to further nicking. The second modification step we implemented was on-bead library preparation which helped in reducing the background and enhancing the signal to noise ratio for the accessible region of the genome. For the UniNicE-seq, after labeling reaction, the biotin-labeled DNA of HCT116 cell was isolated, sonicated, and captured on streptavidin magnetic beads for library preparation and sequencing (Fig. 1b). A slight modification to this protocol by substituting dATP to Texas-Red-dATP allowed both visualization and sequencing (termed as NicE-viewSeq) [8] of the accessible chromatin region (Fig. 1c, please refer to supplementary text 1: detailed stepwise protocol: universal NicE-seq (25000-250000 HCT116 cells), Appendix 2 and 3).

To determine the effect of 5mC incorporation in accessible region for library preparation, we performed UniNicE-seq in the presence of ×10 and ×20 (25 and 50 units) excess Nt. CviPII nicking enzyme. Indeed, neither of the two reaction conditions showed loss of accessible chromatin in HCT116 cells (fraction of reads in called peak regions (FRiP) 0.19–0.14), demonstrating the robustness of 5mdC-labeled accessible chromatin protection against degradation (Supp. Table 1). In a similar reaction condition using 25 and 50 units Nt.CviPII and original NicE-seq protocol, we obtained FRiP score below 0.01 and loss of accessible chromatin peaks (Supp. Table 1). In addition, 2/3rd of peaks overlapped between universal NicE-seq libraries made with ×1, ×10, and ×20 nicking enzymes and signal to noise ratio were comparable, although a reduction in peak height was observed (Fig. 1d, e). These results unequivocally demonstrate the versatility of 5-mdCTP in the reaction to protect accessible regions without titration of the cell to enzyme concentration.

**Fig. 2:**
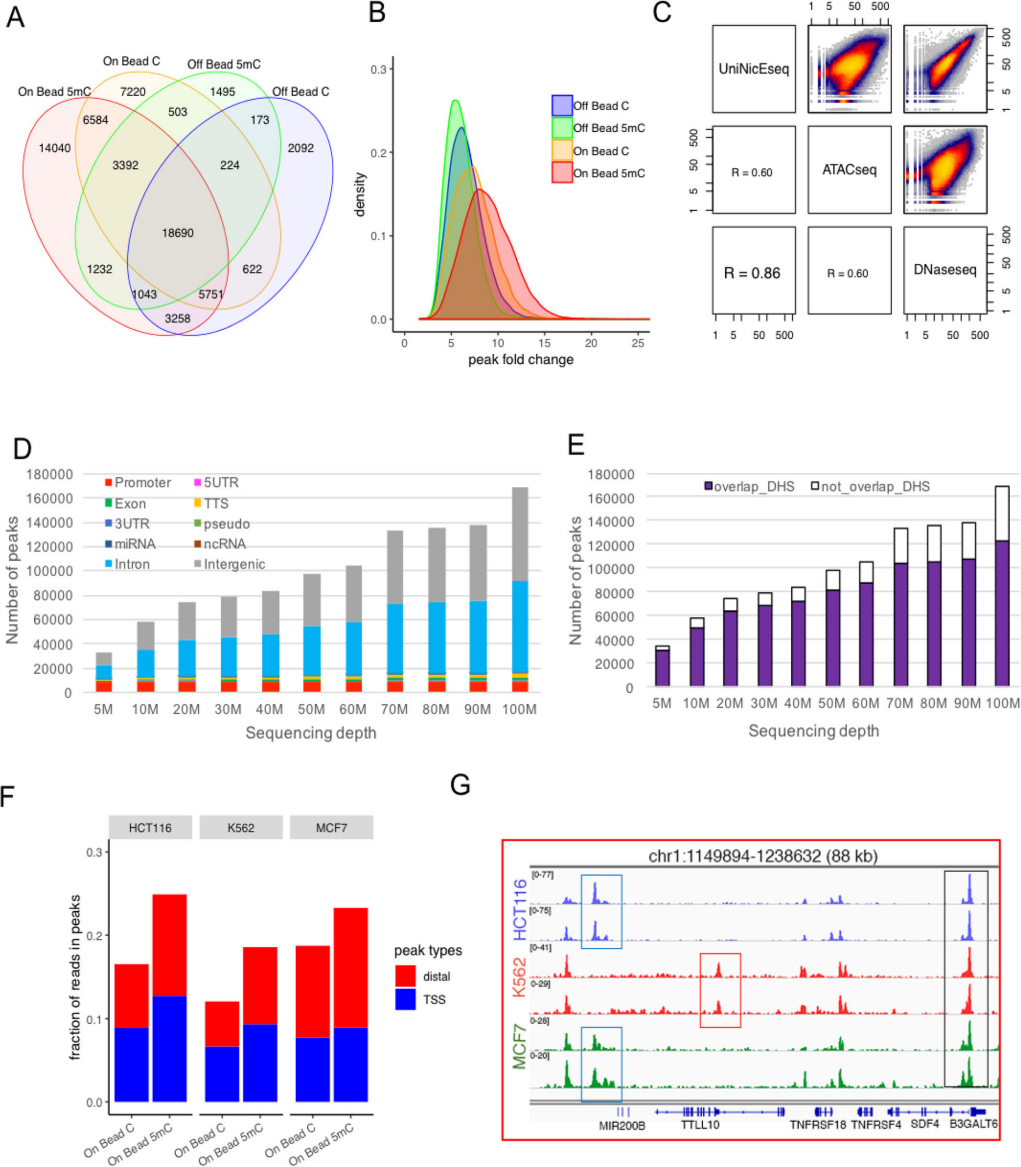
UniNicE-seq optimization and validation. **a** Reaction conditions and library making method comparison. Venn diagram showing the overlap of peaks in various reaction conditions and library making method. **b** Distribution of fold change (FC) values (derived from MACS2) of the accessible chromatin peaks detected by on bead 5mC, on bead C, off bead 5mC, and off bead C methods. Accessible chromatin peaks detected by on bead 5mC (red) showed higher FC values on average than other methods. **c** Pearson correlation of accessible chromatin peak read densities between UniNicE-seq, ATAC-seq, and DNase-seq of HCT116 cells. **d** Distribution of HCT116 UniNicE-seq (On Bead 5mC) peaks in different genomic regions at sequencing depth from 5 to 100 M alignment pairs. **e** Numbers of HCT116 UniNicE-seq accessible chromatin peaks that overlap and not overlap with the reference human DNase I hypersensitivity sites at sequencing depth from 5 to 100 M alignment pairs. **f** Fraction of reads in peaks that map to TSSs (+/−500 bp of TSS) and distal elements (> 500 bp from TSS) from libraries generated using the on-bead UniNicE-seq methods on three human cell lines (HCT116, K562, MCF7). **g** Representative IGV screenshot of the normalized read density of the UniNicE-seq libraries of the three human cell lines, HCT116, K562, and MCF7, from experimental replicates

To assess other improvements in our new method, we compared the number of accessible chromatin peaks detected and peak height, which measures as the ratio of reads within the peak versus background. First, we compared peak numbers between libraries made on beads (on bead) and control (off beads) either with 5-mdCTP or dCTP in the dNTP mix (Supp Fig. 1A). The majority of accessible chromatin peaks are common across these libraries, as shown in the Venn diagram suggesting that our modifications are not changing the distribution of identified regions (Fig. 2a). Furthermore, the signal to noise ratio was improved in libraries made on beads as observed by peak height and distribution of fold change (FC) values (Fig. 2b). For comparison between original NicE-seq (off bead C) and universal NicE-seq (on bead 5mC), the peak fold changes were better (6 vs. 8) and the fraction of reads in the peaks (FRiP) were 0.095 and 0.26, respectively (Supp Fig. 1B).

We further compared the accessible chromatin reaction conditions and reads between UniNicE-seq, ATAC-seq, and DNase-seq of HCT116 using Pearson correlation of peak read densities for data quality evaluation. In terms of data quality, UniNicE-seq and DNase-seq had the highest correlation (Pearsons correlation = 0.86) compared to UniNicE-seq with ATAC-seq (Pearsons correlation = 0.60) or ATAC-seq with DNase-seq (Pearsons correlation = 0.60), demonstrating most DHS (DNase Hypersensitive Sites) are indeed captured in this modified protocol (Fig. 2c). Since nucleosome-free DNA regions differentially affect distant communication in chromatin and accessible chromatins are a hallmark of active gene promoters, we compared accessible chromatin profile and transcription start sites (TSS) in duplicate datasets, across both off-bead and on-bead methods. Accessible chromatin displayed good signal to noise ratio in both on and off bead methods (Supp Fig. 2A). Indeed, all the TSS were enriched with accessible chromatin sequence reads (Supp Fig. 2B, [9]). HCT116 cell nuclei labeling in the presence of 5-mdCTP displayed highest tag densities and higher signal to noise ratio with high degree of correlation ~99% between technical duplicates (Supp Fig. 2A-C). Comparison between UniNicE-seq, ATAC-seq, and DNase-seq peaks of HCT116 showed stronger peak signals in UniNicE-seq sequencing tracts that were derived from equal numbers of unique sequencing reads (Supp Fig. 2D), with a high percentage of common accessible regions between UniNicE-seq and DNase-seq (Supp Fig. 2E). In order to define the accessible chromatin coverage of UniNicE-seq, we also performed sequencing depth analysis and discovered accessible chromatin regions in promoters and other genic regions. Indeed, at a sequencing depth of 5 million reads, we were able to identify most of the HCT116 promoters. At higher sequencing depth of 100 million reads, intronic and intergenic accessible regions were more prominently detectable, although the significance of these regions remains unclear (Fig. 2d). Since UniNiCE-seq and DNase-seq showed a high degree of correlation, we compared both datasets at a sequencing depth of 100 million reads with 10 million read increments and approximately 71% of the UniNicE-seq peaks overlapped with DHS, suggesting strong confidence in the UniNicE-seq method (Fig. 2e). To establish UniNicE-seq as a general protocol, we assayed two additional cell lines, MCF7 and K562, and compared accessible chromatin between libraries made on beads with either with 5-mdCTP or dCTP in the dNTP mix (Supp. Table 2). We calculated fraction of reads in peak (FRiP) analysis of all the peaks generated by UniNiCE-seq to compare with HCT116 cells. The FRiP score was comparable among all three cell lines in both the total number of peaks and promoter peaks, suggesting substitution of 5mC for C and on bead library preparation improves the number of accessible chromatin peaks with an identical sequencing depth of 11 million pair reads (Fig. 2f). As expected, the cell line specific unique and common peaks were also identified in UniNicE-seq (Fig. 2g, Supp Fig. 3) with consistently higher numbers of identified TSS peaks (Supp. Table 2). Taken together, these results demonstrate universal NicE-seq is a superior and robust method than its predecessor NicE-seq protocol to profile accessible chromatin negating the need for enzyme titration and protecting accessible regions for NextGen sequencing.

### Universal NicE-seq of mouse tissue

**Fig. 3:**
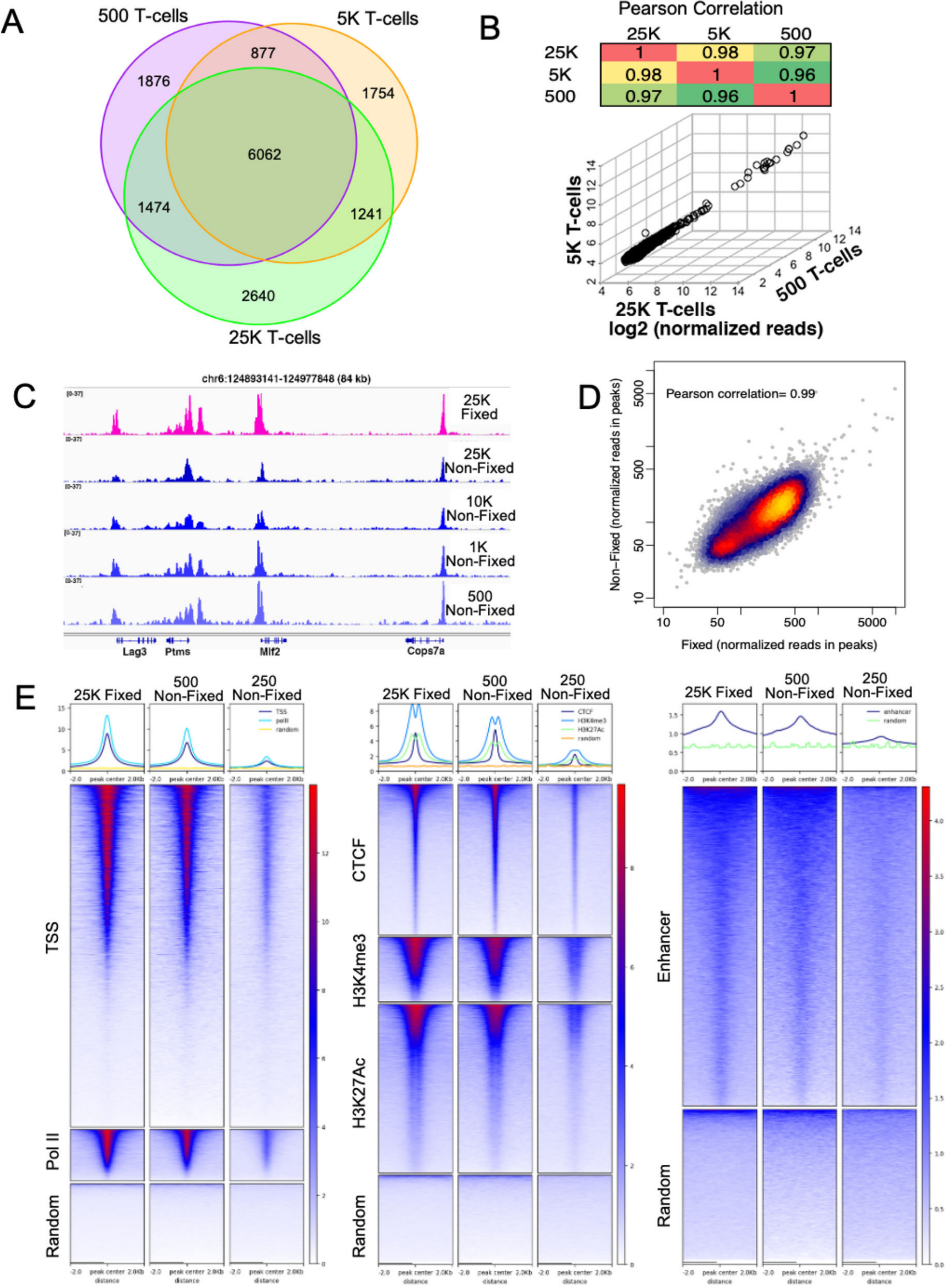
UniNicE-seq of mouse T cells and kidney tissue. **a** Venn diagram demonstrating common accessible regions in mouse T cells with sample size from 25 K, 5 K, and 0.5 K cells. **b** Three-dimensional scatter plot of technical duplicate T cell samples from 25 K, 5 K, and 0.5 K cells. Average log_2_ (normalized reads) in common accessible chromatin peaks between all samples are plotted. A correlation matrix of Pearson values of the common accessible chromatin peaks is shown on top. **c** Representative IGV screenshot of the normalized read density of the UniNicE-seq libraries of mouse kidney nuclei. A comparison between formaldehyde-fixed nuclei and non-fixed nuclei is shown. Non-fixed nuclei numbers were between 100 K to 0.25 K. **d** UniNicE-seq libraries of 25 K fixed and non-fixed mouse kidney cells were down-sized to 21.5 M properly aligned read pairs (after removing mitochondrial reads and PCR duplicates). MACS2 called 40716 narrow peaks from the fixed sample, and 27853 peaks from the non-fixed sample, among them, 15901 are common to both samples. The read density of the 2 libraries in the set of common peaks is highly correlated with a Pearson correlation efficiency of 0.99. **e** Heatmap showing normalized RPKM of 100 K fixed, 0.5 K nonfixed, and 0.25 K non-fixed mouse kidney nuclei at TSS, PolII, and random along with chromatin features including CTCF, H3K4me3, H3K27Ac, and random fragments

We further applied the UniNicE-seq to mouse T cells and kidney tissues. T cells were formaldehyde fixed and serially diluted from 25,000 to 500 cells for UniNicE-seq labeling, and libraries were made in duplicate. The signal to noise ratio of all samples was consistent, and MACS2 peak calling was made with down-sized 13M unique non-mitochondrial reads (Supp Fig. 4A). As expected, the majority of the accessible chromatin peaks were common in all replicates (Fig. 3a). The pairwise total read densities comparison between 500, 5 K, and 25 K cells also displayed a strong correlation of r value between 0.95 and 1 (500 vs. 5 K, *r* = 1; 5 K vs. 25 K, *r* = 0.97; 500 vs. 25 K, *r* = 0.95). Since accessible DNA quantity varied between different cell numbers, we further tested if the read density between the common peaks are similar. For validation, average log_2_ (normalized reads) in 6062 peaks that were detected in all 6 samples were plotted on a 3-dimensional scatter plot. The observation that all data points line up on a straight line in the 3-dimensional space shows that signal in 25000, 5000, and 500 T cells are highly correlated and that there was no loss of signal with decreased numbers of cells (Fig. 3b). A correlation matrix of Pearson values corroborates the above observation (Fig. 3b). A strong correlation was also observed when all reads were compared (Supp Fig. 4B). In addition to mouse T cells, we also interrogated and compared accessible chromatin data from formaldehyde-fixed with unfixed mouse kidney cells. Accessible chromatin from 25 K fixed cells was compared with 25 K, 10 K, 1 K, 500, and 250 unfixed cells. The quality control metrics of UniNicE-seq libraries applied to mouse kidney tissues were good (Supp. Table 3). The peak numbers between fixed and unfixed cells were similar along with the signal to noise ratio between 25000 and 500 cells (Fig. 3c, Supp. Table 3). We also observed that the nonfixed high number kidney nuclei (> 1 K) would form clumps and make the NicE-seq labeling reaction inefficient resulting in a decrease in FRiP score (Supp. Table 3). Direct comparison between normalized total reads of common accessible region peaks of 25000 fixed and non-fixed cells displayed good correlation (*P* = 0.98), suggesting both samples are compatible with the UniNicE-seq protocol, and is robust without the enzyme titration requirement for low cell numbers (Fig. 3d). Secondly, we extracted previously published genomic features such as TSS, Pol II, CTCF, and histone modification sequences from universal NicE-seq reads for analysis. Heatmap analyses showing normalized RPKM at TSS and enhancers of 24,920 mouse Ref-seq genes, and the surrounding 2 kb region displayed the central accessible region. Further comparison with mouse kidney H3K4me3, H3K27Ac, PolII, and CTCF ChIP-seq data demonstrated an enrichment of accessible regions as expected (Fig. 3e). We further compared data sets from the UniNicE-seq method generated from 25 K-fixed mouse kidney cells and 0.25 K non-fixed cells with Omni-ATAC-seq (improved ATAC-seq, 7) and DNase-seq datasets. In experiments involving UniNicE-seq and unfixed 500 cells and Omni-ATAC-seq and 50 K cells, 65% of the peaks from UniNicE-seq were common with Omni-ATAC-seq. (Supp Fig. 5A). Analysis of tag density and peak width as well as FRiP score comparison suggested that UniNicE-seq on 500 unfixed mouse kidney cells yielded high quality accessible chromatin data (Supp Fig. 5B, C). TSS, PolII, and active enhancer and chromatin marks were also prominent with 500 kidney cells (Supp Fig. 5D, E). These results show that genes with active promoters and enhancers are captured by UniNicE-seq as open chromatin regions, regardless of fixation and with small cell number from tissue samples.

### Universal NicE-seq of human 5–10-μm frozen tissue sections

Clinical samples from patients are often limited and increasingly obtained from fresh biopsies. Routinely, 5–10-μm tissue sections are used for immunopathological and other staining analysis. Currently, the ATAC-seq method for chromatin accessibility studies requires 50,000 purified nuclei from the patient brain and cartilage tissue samples [7, 10]. Indeed, 20 mg of brain tissue samples were used from post-mortem human brain samples for nuclei preparation [7]. However, obtaining large tissue volume from live patients is inconvenient, and often donor samples are limited. Therefore, we attempted UniNicE-seq using single-frozen 5–10-μm tissue sections, mimicking biopsy samples. The protocol was slightly modified for this application, with a formaldehyde fixation step of the tissue section prior to the labeling reaction on the slide. The fixing ensured the attachment of tissue section for on-slide labeling reaction and subsequent washing steps. We then applied the UniNicE-seq protocol to make accessible chromatin libraries from tissue sections. Accessible chromatin regions were revealed, as expected, between two different lung tissue sections (Supp Fig. 6A, Supp. Table 4). Total read counts between these two tissue sections displayed a strong correlation *r* = 0.97 (Supp Fig. 6B). Similarly, all common accessible peak reads between both tissue sections also were highly correlated (*r* = 0.97) demonstrating the high quality of common accessible regions (Supp Fig. 6C).

**Fig. 4:**
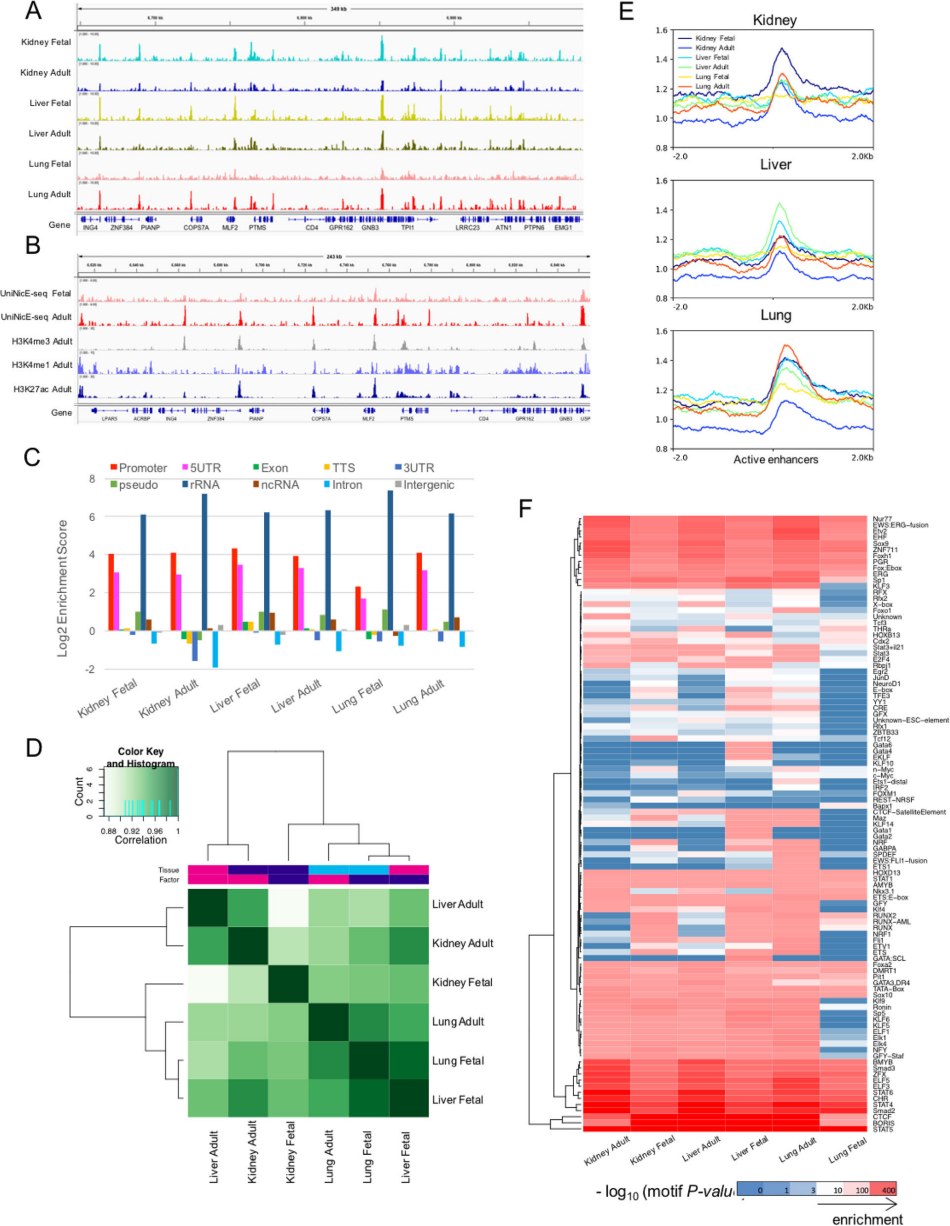
UniNicE-seq of 5-10 μm human frozen tissue sections and genomic features analysis. **a** Representative IGV screenshot of the normalized read density of the UniNicE-seq libraries of human fetal and adult tissue sections derived from the kidney, liver, and lung. **b** Representative IGV screenshot of the normalized read density of the UniNicE-seq libraries of fetal and adult lung tissue sections with ChIP-seq chromatin marks of adult tissue for H3K4me3, H3K4me1, and H3K27Ac. **c** Enrichment of UniNicE-seq peaks with various genomic features. **d** Correlation analysis of UniNicE-seq peaks of human fetal and adult tissues. **e** Profile plot of UniNicE-seq open chromatin signals (peak density) of the human tissue samples in the kidney, liver, and lung tissue-specific active enhancers (AE). **f** Heatmap representing the enrichment of consensus TF-binding motifs identified in each tissue sample near the UniNicE-seq peaks. Both of the TF binding motifs and the samples are organized by the unsupervised k-means clustering method. The *p* values of e−6 were considered for the cluster analysis

Following the reproducibility of accessible chromatin of lung tissue, we then applied the UniNicE-seq protocol to make accessible chromatin libraries from human adult and fetal tissue including the liver, lung, and kidney; pooled both replicates and down-sized the reads; and compared accessible chromatin landscape among them to examine developmental and tissue-specific differences of accessible chromatin. Indeed, accessible chromatin regions were revealed, as expected, across all tissue sections (Fig. 4a). Active enhancer and promoter histone marks, particularly H3K4me1, H3K27Ac, and H3K4me3 coincided with all accessible regions identified by UniNicE-seq and correlated with curated promoters in human adult lung tissue compared to fetal lung tissue (Fig. 4b). Across all tissues, accessible chromatin was enriched in promoter, 5’UTR, and rRNA genic regions demonstrating commonality further supporting our method is compatible with the identification of open chromatin peaks in tissue sections (Fig. 4c).

Cells undergo transcriptional changes during differentiation, especially the acquisition of tissue-specific enhancers. Therefore, we profiled enhancers in fetal and adult lung tissues using known histone marks associated with enhancers. However, in the fetal and adult lung, there was a marked difference in the accessibility of promoter and 5’UTR regions (Fig. 4c). When we compared adult vs. fetal tissues, a varying degree of correlation was observed among all 3 different tissue types (Fig. 4d). Accessibility of active enhancer elements in the kidney was more enriched in the fetal tissue compared to liver enhancers that predominated in the adult tissue. As expected, active enhancers in adult lung tissues were more enriched compared to the fetal tissue suggesting a developmental cue for enhancer activation post birth (Fig. 4e). Similarly, transcription factor consensus binding site near the UniNicE-seq-derived accessible chromatin-binding region displayed varying degrees of similarity and a contrast between fetal and adult tissues, with fetal tissue demonstrating developmental and functional programming of lung tissue (Fig. 4f). Principal component analysis of read counts between fetal and adult tissue accessible chromatin displayed close similarity in the liver compared to the kidney and lung tissue (Supp Fig. 6D). However, transcription start sites were always accessible in both fetal and adult tissue to varying degrees (Supp Fig. 6E).

### One-tube universal NicE-seq protocol of human 5–10-μm FFPE tissue sections

**Fig. 5:**
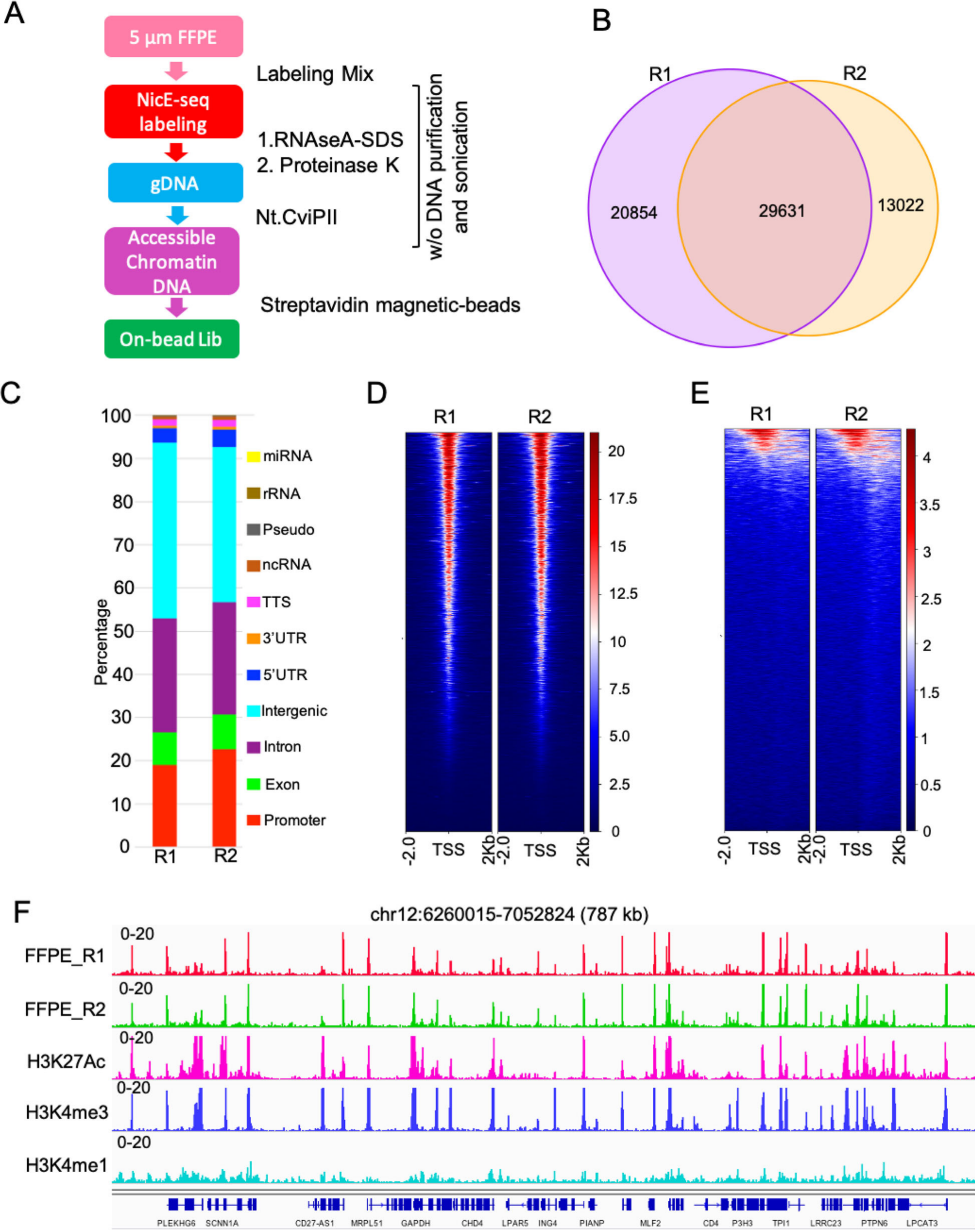
One tube UniNicE-seq of 5–10-μm human liver FFPE tissue sections. **a** Workflow diagram of one tube universal NicE-seq. (**b**) Venn diagram demonstrating common accessible regions in two different human 5–10-μm liver normal FFPE tissue sections designated as R1 and R2. **c** Peak annotation of accessible peaks of liver FFPE tissue section showing the genomic feature distribution. **d** Heatmap and signal intensity profile plot of TSS (that includes ±2 Kb of flanking region) in liver FFPE tissue section. **e** Heatmap and signal intensity profile plot of active enhancer elements (that includes ±2 Kb of flanking region) in liver FFPE tissue section. **f** Representative IGV screen shot of accessible chromatin peaks of two liver FFPE tissue section and histone marks, H3K27Ac, H3K4me3, and H3K4me1

Change in chromatin accessibility underlie various diseases including cancers and are often available as FFPE samples. Therefore, we modified universal NicE-seq to be compatible with FFPE tissue sections. The major goal was to develop the reaction protocol that will negate the DNA purification and sonication step and will directly feed into NGS library preparation (Fig. 5a).

We introduced a few notable changes. For removal of paraffin, we introduced warm paraffin oil wash in place of organic solvent, which is flammable and would need special handling. After paraffin oil-based removal of paraffin, the tissue sections were hydrated and equilibrated in PBS for compatibility of the labeling reaction. Once the labeling reaction was over, RNaseA was added and crosslinking was reversed at 65 °C incubation. The reaction mix was treated with proteinase K for removal of any protein to enrich DNA in the tube. In the next step, proteinase K was heat-inactivated and nicking enzyme Nt.CviPII was added again to the reaction. Since Nt.CviPII is sensitive to newly labeled accessible chromatin incorporated with 5mC, it would digest away all other DNA of the genome, thus enriching only accessible regions. These accessible DNA fragments were captured on streptavidin beads for on-bead NGS library preparation and sequencing (Supplementary text 1: Please refer to supplementary text 1 and appendix 4: detailed stepwise protocol: universal NicE-seq (25000-250000 HCT116 cells).

To demonstrate the proof of principle, two different human liver FFPE samples were profiled for accessible chromatin studies (Supp. Table 5). Both tissue sections displayed 60–70% common accessible regions and a strong correlation of read counts (Fig. 5b, Supp Fig. 7). Most accessible regions were enriched in the promoter, exon, intron, and intragenic regions as expected (Fig. 5c–e). IGV browser display showed an accessible chromatin peak correlation with active chromatin marks, H3K27ac, and H3K4me3 along with active enhancer mark, H3K4me1 (Fig. 5f). Therefore, we concluded that universal NicE-seq works well in a single-FFPE tissue section.

## Discussion

In summary, we demonstrated the ease of application of UniNicE-seq to a variety of human cell lines, mouse tissues and human organ-derived tissue sections from both frozen-fixed and FFPE samples for obtaining chromatin accessibility information. Two protocol modifications, 5-mdCTP during labeling and an on-bead capture of labeled DNA and library preparation, led to enhance robustness without the need for significant optimization. This improved UniNicE-seq method employs a single protocol without any enzyme tittering for optimization of the reaction time to enable accessible chromatin mapping in both unfixed and formaldehyde-fixed cells, and 5–10-μm frozen tissue sections in contrast with Tn5 transposon-based method where the transposon simultaneously fragment and tag the unprotected regions of DNA and often needs labor-intensive optimization, although the addition of 10–20 fold excess enzymes resulted in lower peak height in our experiments. We suspect the excess nicking enzyme remains bound to accessible DNA with 5mC/bt-mC and interferes with PCR and library making steps. However, more studies needed to know the true nature of this observation (Fig. 1d). In our test conditions, universal NicE-seq, DNase seq, and ATAC-seq majority accessible chromatin peaks were common. Since our method uses CCD sequence-specific nicking enzyme Nt. CviPII, it is plausible that our peaks may incur GC bias. To compare GC content of all the HCT116 peaks, we analyzed 15 M unique alignment pairs from UniNicE-seq, ATAC-seq, and DNase-seq with randomly sampled genome background. The boxplot showed that all the 3 methods indeed have a bias toward high GC content comparing to the genome background. This is due to the fact that many open chromatin regions are in the promoter and enhancer of the genes that are CpG rich or overlap with the CpG islands. When comparing between all three different methods, we were not able to find a significant difference, confirming that our UniNicE-seq does not result in additional bias from the CCD recognition sequence of the nick enzyme (Supp Fig. 8). Furthermore, we have demonstrated that universal NicE-seq is compatible with FFPE tissue sections without DNA purification and sonication. This is particularly important since small cell numbers or limited biological material do not yield large amounts of DNA, and any purification step would result in loss of the DNA. Similarly, sonication-mediated DNA sizing on limited amounts of DNA is not advisable. Therefore, one-tube universal NicE-seq will simplify the workload and be more consistent.

Till date, ATAC-seq has not been shown to work on the FFPE tissue section. However, it may be noted that the integrity of DNA is essential for any accessible chromatin mapping in FFPE tissue samples, since most accessible regions are ~500 bp length. Similarly, for one-tube universal NicE-seq, the SDS detergent concentration may play a role in subsequent Nt. CviPII digestion of unmodified DNA. Thus, UniNicE-seq offers one step “labeling and protection” of accessible chromatin. Furthermore, UniNicE-seq of 5–10-μm frozen tissue and FFPE sections does not need nuclei purification, thus have the potential to parallel process multiple tissue sections. Indeed, in a recent pan-cancer accessible chromatin studies using ATAC-seq, the authors have used 20 mg of cancer tissues to isolate nuclei for library preparation [11]. These features demonstrate that UniNicE-seq is a robust method for use with frozen and fixed developmental tissue samples that could be extended to include clinical tissue samples which are currently difficult to obtain in large quantity for chromatin accessibility studies.

## Methods

### Cell culture

HCT116 (ATCC, USA) cells were cultured in McCoy’s 5A media supplemented with 10% fetal bovine serum. MCF7 and HeLa cells (ATCC, USA) were cultured in DMEM plus 10% FBS.

### Accessible or open chromatin labeling of cells and tissue sections

Twenty-five thousand HCT116 cells were used for routine library construction. Cells were cross-linked using 1% formaldehyde for 10 min at room temperature and quenched by using 125 mM glycine. Nuclei were isolated by incubating the cross-linked cells in cytosolic buffer (15 mM Tris-HCl pH 7.5, 5 mM MgCl_2_, 60 mM KCl, 0.5 mM DTT, 15 mM NaCl, 300 mM sucrose, and 1% NP40) for 10 min on ice with occasional agitation. Nuclei were precipitated by spinning at 1000 × *g*, 4 °C for 5 min and supernatant were discarded. The nuclei pellet was resuspended in 100 μl of ×1 PBS. Open chromatin DNA was labeled with biotin by incubating the nuclei in the presence of 2.5 U of Nt.CviPII (NEB, R0626S), 50 U of DNA polymerase I (NEB, M0209S) and 30 μM of each dGTP and dTTP, 24 μM of 5-mdCTP (NEB, NO356S) and dATP 24 μM plus 6 μM of biotin-14-dATP (Invitrogen, 19524016) and 6 μM of biotin-14-dCTP (Invitrogen, 19518018) at 37 °C for 2 h with occasional mixing. The labeling reaction was stopped with 20 μL of 0.5 M EDTA, and 2 μg of RNase A was added to the labeling reaction and incubated at 37 °C for 0.5 h to digest RNA. The DNA was isolated using phenol-chloroform extraction and ethanol precipitation method or Qiagen spin column (Qiagen, USA). For NicE-seq libraries using 25 or 50 units of enzymes, the reaction conditions were identical except that the incubation was at 800 RPM at 37 °C for maximal NicE-seq labeling.

For tissue samples, mouse T cells, human liver, and mouse kidney cells, nuclei were prepared and formaldehyde-fixed before labeling reaction, as described above. In experiments involving unfixed tissue nuclei, the nuclei suspension was in ×1 PBS before labeling.

Frozen tissue sections of lungs on slides were procured from US Biomax, Inc. ( HuFTS251). For frozen 5–10-μm tissue section fixing and labeling, the slides were treated with 1% formaldehyde for 10 min at room temperature and quenched by using 125 mM glycine. After ×1 PBS wash, the slide was treated with cytosolic buffer (15 mM Tris-HCl pH 7.5, 5 mM MgCl_2_, 60 mM KCl, 0.5 mM DTT, 15 mM NaCl, 300 mM sucrose, and 1% NP40) for 10 min at 4 °C. 200 μl of the labeling buffer was placed on the tissue section, and the slide was transferred to a humidified chamber at 37 °C for 2 h. Please refer to supplementary text 1, appendix 1 and 3: detailed stepwise protocol: universal NicE-seq (25000-250000 HCT116 cells).

### Accessible or open chromatin labeling of FFPE sections

FFPE slides of the liver sample were custom ordered from BioChain, USA, with 24–48 h of formalin fixation. Slides were mounted with 5 μm sections. The slide was incubated at 52 °C for 20 min with mineral oil (Sigma, USA), followed by gradual ethanol wash (100%, 90%, 80%, 70% ethanol) in jars at room temp for 5 min each step. The final wash was in milli-Q water for 2 min. Then, the slides were incubated in PBST buffer (×1 PBS in 0.1% Tween 20) at 65 °C for 1 h, and incubated again in ×1 PBST buffer containing protease inhibitor (Sigma, USA) at room temperature for 10 min, washed by ×1 PBS buffer, and dried at the room temperature. The slides were rehydrated with ×1 PBS buffer and treated with the cytosolic buffer for 10 min at 4 °C. Cytosolic buffer was washed away, and the NicE-seq labeling reaction was identical to the frozen tissue section procedure.

For FFPE samples, after the labeling reactions, the labelled nuclei sample is collected in 200 μl of ATL buffer (Qiagen), 20 µl (200 units) of proteinase K (NEB, USA) was added, and the reaction was incubated at 65 °C for o/n for decrosslionking. Proteinase K was inactivated by incubating the reaction tube at 95 °C for 2 min. We next added 2.5 units of Nt.CviPII, 50 µl of NEB buffer 2 and adjusted the reaction volume to 500 µl with water and incubated for 16 h at 37 °C. Nt. CviPII enzyme in the reaction was heat-inactivated at 65 °C for 15 min. These processes ensured that accessible chromatin fragments in the solution for streptavidin magnetic bead enrichment as described below. For formaldehyde fixed cells and tissue nuclei, the decrosslinking step was performed by adding 20 µl of 20% SDS, 20 µl of proteinase K and the reaction was incubated at 65˚C o/n, and processed as described above. Please refer to supplementary text 1, appendix 4 detailed stepwise protocol: universal NicE-seq (25000-250000 HCT116 cells).

### Selective enrichment of labeled accessible/open chromatin

The isolated genomic DNAs (~200 ngs/25 K cell) were sonicated into 150 bp fragments (Covaris) and the entire reaction product was mixed with 20 μL of Streptavidin magnetic beads (Invitrogen 65001, blocked using 0.1% cold fish gelatin in 1 × PBS overnight at 4 °C) in 1 mL of B&W buffer (10 mM Tris-HCl pH 8.0, 1 mM EDTA, 2 M NaCl). Biotin-labeled open chromatin DNA was captured by streptavidin at 4 °C for 2 h with end-over-end rotation. The beads were washed four times with B&W buffer plus 0.05% of Triton X-100 followed by one-time wash with TE. The beads were resuspended in 50 μL of TE. The DNA was end-repaired, washed twice with B&W buffer plus 0.05% of Triton X-100, dA-tailed, and washed with B&W buffer plus 0.05% of Triton X-100. And finally, NEB Illumina adaptor (NEB, E7370S) was ligated and washed twice with B&W buffer plus 0.05% of Triton X-100. A final wash of the bead-bound DNA was performed with TE, and the bound DNA was resuspended with 20 μl of TE. Ten to 20 μL of bound DNA was used for library amplification using PCR (NEB, E7370S). Routinely, 8–10 PCR cycles were used to generate enough amount of library DNA for sequencing. Low input cell numbers between 250 and 500, the library was amplified 12–13 cycles. The library was examined and quantitated with a high-sensitive DNA chip (Agilent, 5067-4627).

### Bioinformatics analyses

#### Data processing and peak calling

Adaptor and low-quality sequences were trimmed from paired-end sequencing reads using Trim Galore (http://www.bioinformatics.babraham.ac.uk/projects/trim_galore/) with the following setting: --clip_R1 4 --clip_R2 4 --three_prime_clip_R1 4 --three_prime_clip_R2 4. Trimmed read pairs were mapped to the reference genome (mouse: mm10; human: hg38) using Bowtie2 [12] with the following arguments: --dovetail --no-unal --no-mixed --no-discordant --very-sensitive -I 0 -X 1000. Prior to peak calling, PCR duplicates and mitochondrial reads were removed and only properly aligned read pairs were used for peak calling with MACS2 [13] using ‘macs2 callpeak -f BAMPE -m 4 100 --bdg –SPMR’.

In order to compare NicE-seq data generated from different protocols, different numbers of input cells, and different samples or compare NicE-seq to other open chromatin mapping methods (i.e., ATAC-seq, DNase-seq), we down-sized the mapped reads from different experiments to the same number of mapped fragments (after excluding PCR duplicates and mitochondrial reads) through random sampling. Peaks were called using the same parameter with MACS2, as mentioned above. Bigwig files of normalized reads per million read in 10 bp non-overlapping windows across the genome were displayed in the Integrated Genomics Viewer [14]. All analyses were performed after removing ENCODE blacklists.

#### Fraction of reads in peaks (FRiP)

The FRiP score was calculated using the deepTools plotEnrichment function [15]. Called peaks were classified into 2 groups: TSS peaks if they overlap with +/−500 bp from annotated TSSs (based on NCBI RefGene annotation), and distal peaks if otherwise. Correspondingly, reads that overlapped with the TSS peaks by at least 1 base were marked as “TSS” reads. Reads that overlapped with distal peaks were marked as “distal” reads. Reads that do not overlap with any called peaks were marked as “reads not in peaks.”

#### Peak overlap analysis

Peaks called from different experiments were compared using the Bedtools [15]. First peaks from all the samples are concatenated. Peaks that have at least one base pair overlapping are considered associated and are merged to form a union peak set. Then, peaks of individual samples were compared to the union set and were marked as either “unique” or “common.” Last, the numbers of “unique” and “common” peaks were summarized from all the samples and were used to make Venn Diagrams in R.

#### Correlation analysis

Correlation analysis of UniNicE-seq open chromatin signals was performed with the DiffBind [16] package in R and deepTools [15] using two methods: occupancy (peak overlap)-based method uses peak overlapping states and affinity (normalized read density)-based method. The occupancy-based method determines the correlation coefficients based on the numbers of unique peaks and overlapping peaks. The affinity-based method first determines the number of normalized reads that overlap with a set of consensus peaks for individual samples and then calculates the Pearson correlation based on the normalized read count matrix. Correlation heat maps were generated using both occupancy and affinity methods with DiffBind and deepTools. PCA plots were generated from the normalized read count matrix by the affinity method.

#### Peak annotation and gene/genome ontology analysis

Functional annotation of called peaks was performed with HOMER [17] annotatePeaks.pl. After associating peaks with nearby genes and assigning peaks to different genomic features (e.g., promoter, exon, CpG islands, repetitive elements, etc.), we also conducted Gene Ontology enrichment analysis for selected sets of UniNicE-seq peaks (e.g., tissue-specific peaks) and tested for enrichment of UniNicE-seq peaks in associated genomic features with HOMER.

#### Peak profile analysis in epigenetic relevant regions

To investigate enrichment of open chromatin signals over sets of genomic regions with epigenetic significance, we first calculated normalized read coverage (number of reads normalized by the scaling factor of Reads Per Kilobase per Million mapped reads (RPKM)) in tiling bins of 100 bp across the entire genome from the bam file of properly aligned fragments (excluding PCR duplicates and mitochondrial reads) using the deepTools [15]. Then, heatmaps and profile plots were generated based on the normalized read coverage per 100 bp-bin over sets of genomic features of interest (TSS, enhancer, CTCF binding sites, RNA polymerase II binding sites) and the surrounding +/−2 Kb regions. As a control, the normalized coverage of a set of randomly sampled genomic regions was also plotted in the same way.

#### External datasets

TSS of mouse (mm10) and human (hg38) genomes were extracted from the NCBI RefGene gene table downloaded from the UCSC Table Browser. ChIP-seq datasets of cell-specific and tissue-specific CTCF binding, RNA polymerase II binding, and histone marks (H3K27ac, H3K4me1, H3K4me3) were downloaded from the mouse and human Encyclopedia of DNA Elements (ENCODE) projects (Supp. Table 6). Human liver tissue-specific enhancers were acquired from the TiED database (http://lcbb.swjtu.edu.cn/TiED/) and EnhancerAtlas [18]. The original hg19 genome coordinates were converted to hg38 using the LiftOver tool.

ATAC-seq (SRX2717891 & SRX2717892) and OmniATAC-seq (SRX2717893 & SRX2717894) datasets of the mouse kidney were downloaded from the NCBI SRA database. The DNase-seq dataset of mouse kidney (ENCSR000CNG) was acquired from the ENCODE project. The human HCT116 ATAC-seq and WGBS datasets were downloaded from the NCBI GEO database (ATAC-seq: GSE101966 and WGBS: GSE97889). The DNase-seq dataset was acquired from the ENCODE project (ENCSR000ENM).

#### Data availability

All the UniNicE-seq data generated in this study are deposited in NCBI Gene Express Omnibus (GEO) under the accession GSE140276.

## Supplementary information

**Additional file 1: Supp Fig. 1** Optimization of universal NicE-seq (A) A schematic diagram of accessible chromatin labeling using dCTP or 5-mdCTP in the labeling reaction along with biotinylated-dCTP in the nucleotide mix. On-bead and off-bead represented presence of streptavidin magnetic beads for DNA capture and library preparation. (B) FRiP comparison between all 4 methods generated library that map to TSSs (+/-500 bp of TSS) and distal elements (>500 bp from TSS) from HCT116 cells. C and 5mC represents use of dCTP and 5-dCTP in the reaction mix. **Supp Fig. 2:** Optimization of accessible chromatin sequencing and comparison between UniNicE-seq, ATAC-seq and DNase-seq. (A) IGV screen shot of the normalized read density of the four NicE-seq conditions in HCT116 cells. (B) Distribution of the number of normalized HCT116 NicE-seq reads at transcription start sites (TSS) of human genes and the surrounding 2 Kb (- and +) regions. (C) Pearson correlation of normalized read densities in UniNicE-seq peaks of the 2 technical replicates in HCT116 demonstrating reproducibility. (D) IGV screen shot of the normalized read density of UniNicE-seq (top track), ATAC-seq (middle track) and DNase-seq (bottom track) in HCT116 (F) Overlap of HCT116 peaks called from 15 M unique alignments using UniNicE-seq, ATAC-seq and DNase-seq. **Supp Fig. 3:** Venn Diagram showing common and cell-type specific UniNicE-seq peaks between the three cell types. (A) HCT116, K562 and MCF7 accessible chromatin regions were analyzed. Peaks are called from 11 million random sampled deduplicated alignment pairs. **Supp Fig. 4:** UniNicE-seq of mouse T cells cells. (A) IGV screen shot of the normalized read density of the technical duplicates of UniNicE-seq libraries of HCT116 cells at different cell numbers. (B) Pairwise comparison between all Universal NicE-seq reads between different T cell numbers from 500, 5 and 25 K. Pearson’s correlation is indicated. **Supp Fig. 5:** Comparison between UniNicE-seq, ATAC-seq, Omini ATAC-seq and DNase-seq of mouse kidney cells. (A) Venn diagram of accessible chromatin regions derived from UniNicE-seq, ATAC-seq, Omini ATAC-seq and DNase-seq of mouse kidney cells. (B) Distribution of fold change (FC) values (derived from MACS2) of the common accessible chromatin peaks of UniNicE-seq, ATAC-seq, Omni ATAC-seq and DNase-seq. (C) FRiP score of UniNicE-seq 25 K, 0.5 K and 0.25 K compared with data obtained from ATAC-seq and OmniATAC-seq using 50 K cells, and DNase-seq. (D) Heatmap showing comparison of normalized RPKM of 25 K fixed, 0.5 K nonfixed and 0.25 K mouse kidney UniNicE-seq, 50 K omni ATAC-seq, 50 K ATAC-seq and DNase-seq data at TSS, PolII and random. (E) Similar comparison like (D) along with chromatin features including CTCF, H3K4me3, H3K27Ac and random fragments. **Supp Fig. 6:** Comparison between accessible chromatin sequences two liver FFPE tissue section (A) Venn diagram demonstrating common accessible regions in two different human 5-10 μm lung normal tissue sections. (B) Pearson’s correlation analysis of total reads between two different human 5-10 μm lung normal tissue sections. (C) Pearson’s correlation analysis of common reads between two different human 5-10 μm lung normal tissue sections demonstrating quality of accessible peaks. (D) Principle Component Analysis and heat map of TSS across fetal and adult tissue for normalized read density of the consensus peaks between the samples from fetal and adult tissue. (E) Heat map of TSS (-/+ 2 kb) between various tissue samples. **Supp Fig. 7:** UniNicE-seq of normal human liver FFPE tissue section**.** Pearson’s correlation analysis by pairwise comparison between two different liver samples, R1 and R2. **Supp Fig. 8:** GC content of HCT116 peaks called from 15 M unique alignment pairs using UniNicE-seq, ATAC-seq and DNase-seq. The last box represents the GC content of random genomic regions sampled from the human reference genome (hg38).

**Additional file 2: **Supplementary Table 1: HCT116 UniNicE-Seq matrix for library with different amounts of enzyme. Note 25 and 50 U NicE-seq labeling reactions were incubated at 37 °C at 800 RPM. Supplementary Table 2: Quality control metrics of UniNicE-seq libraries applied to two human cell lines K562 and MCF7 in comparison to libraries made on and off beads with either with 5mdCTP or dCTP in the dNTP mix. We examined percentage of mitochondrial reads (“%mito”), number of total peaks and promoter peaks (+/- 500 bp of TSS) and enrichment of signal at TSSs (“FRiP (TSS peaks)”). Two technical replicates were conducted for each sample. All the values were calculated from a subsample of 11 million de-duplicated alignment pairs. Supplementary Table 3. Quality control metrics of UniNicE-seq libraries applied to mouse kidney tissues. 25 K fixed cells were compared with 25 K, 10 K, 1 K, 0.5 K and 0.25 K unfixed cells. Supplementary Table 4. **a.** Quality control metrics of UniNicE-seq libraries applied to human adult lung tissues. **b**. Quality control metrics of UniNicE-seq libraries applied to different human adult and fetal tissues.^*^ *Here the replicates are merged together and downsized to 50 M aligned pairs for the downstream analysis. Supplementary Table 5. Quality control metrics of UniNicE-seq libraries applied to human FFPE liver tissue sections. Supplemental Table 6. External ChIP-seq data sets of various human and mouse tissue and cell types in this work.

**Additional file 3: Supplementary text 1.** Detailed stepwise protocol: universal NicE-seq (25000-250000 HCT116 cells).

## Data Availability

All the UniNicE-seq data generated in this study are deposited in NCBI Gene Express Omnibus (GEO) under the accession GSE140276. A step-wise protocol is included in the supplementary text 1. Further details can be obtained from the corresponding author.

## Abbreviations

FFPE: Formalin-fixed paraffin-embedded
NicE-seq: Nicking enzyme-assisted sequencing
UniNicE-seq: Universal nicking enzyme-assisted sequencing
DNase-seq: DNase hypersensitive region sequencing
ATAC-seq: Assay for Transposase-Accessible Chromatin using sequencing
5-mdCTP: 5-Methyldeoxycytidine triphosphate
dNTP: Deoxynucleoside triphosphate
dATP: Deoxyadenosine triphosphate
dCTP: Deoxycytidine triphosphate
FRiP: Fraction of reads in peaks
DNA Pol I: DNA polymerase I
DHS: DNase hypersensitive sites
TSS: Transcription start sites
RPKM: Reads per kilobase of transcript, per million mapped reads
5’UTR: 5’ Untranslated region
rRNA: Ribosomal RNA
PBS: Phosphate-buffered saline
PCR: Polymerase chain reaction
CTCF: CCCTC-binding factor
ENCODE: Encyclopedia of DNA Elements
