# Supplementary figures and images for: Universal NicE-seq for high-resolution accessible chromatin profiling for formaldehyde-fixed and FFPE tissues
Source: Clin Epigenetics. 2020 Sep 22;12:143. doi: 10.1186/s13148-020-00921-6 (PMC7507628; doi:10.1186/s13148-020-00921-6)

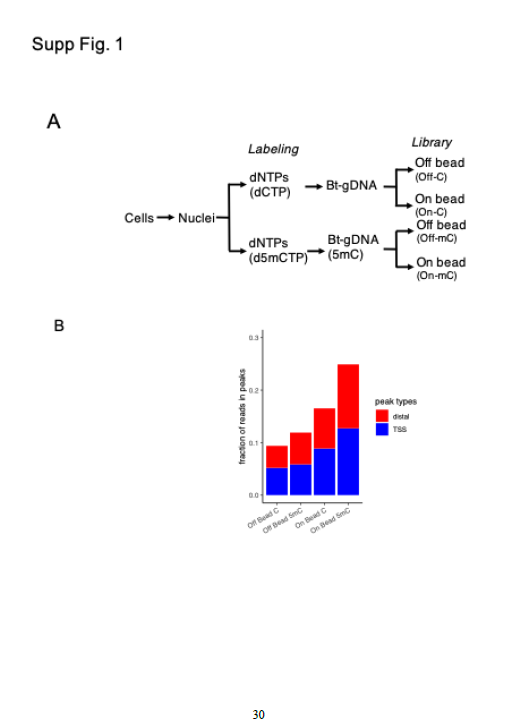

Supplement: Supplementary file 1 — Additional file 1: Supp Fig. 1 Optimization of universal NicE-seq (A) A schematic diagram of accessible chromatin labeling using dCTP or 5-mdCTP in the labeling reaction along with biotinylated-dCTP in the nucleotide mix. On-bead and off-bead represented presence of streptavidin magnetic beads for DNA capture and library preparation. (B) FRiP comparison between all 4 methods generated library that map to TSSs (+/-500 bp of TSS) and distal elements (>500 bp from TSS) from HCT116 cells. C and 5mC represents use of dCTP and 5-dCTP in the reaction mix. Supp Fig. 2: Optimization of accessible chromatin sequencing and comparison between UniNicE-seq, ATAC-seq and DNase-seq. (A) IGV screen shot of the normalized read density of the four NicE-seq conditions in HCT116 cells. (B) Distribution of the number of normalized HCT116 NicE-seq reads at transcription start sites (TSS) of human genes and the surrounding 2 Kb (- and +) regions. (C) Pearson correlation of normalized read densities in UniNicE-seq peaks of the 2 technical replicates in HCT116 demonstrating reproducibility. (D) IGV screen shot of the normalized read density of UniNicE-seq (top track), ATAC-seq (middle track) and DNase-seq (bottom track) in HCT116 (F) Overlap of HCT116 peaks called from 15 M unique alignments using UniNicE-seq, ATAC-seq and DNase-seq. Supp Fig. 3: Venn Diagram showing common and cell-type specific UniNicE-seq peaks between the three cell types. (A) HCT116, K562 and MCF7 accessible chromatin regions were analyzed. Peaks are called from 11 million random sampled deduplicated alignment pairs. Supp Fig. 4: UniNicE-seq of mouse T cells cells. (A) IGV screen shot of the normalized read density of the technical duplicates of UniNicE-seq libraries of HCT116 cells at different cell numbers. (B) Pairwise comparison between all Universal NicE-seq reads between different T cell numbers from 500, 5 and 25 K. Pearson’s correlation is indicated. Supp Fig. 5: Comparison between UniNicE-seq, ATAC-seq, Omini A [file 13148_2020_921_MOESM1_ESM.zip › Additional file Fig s1.PNG]

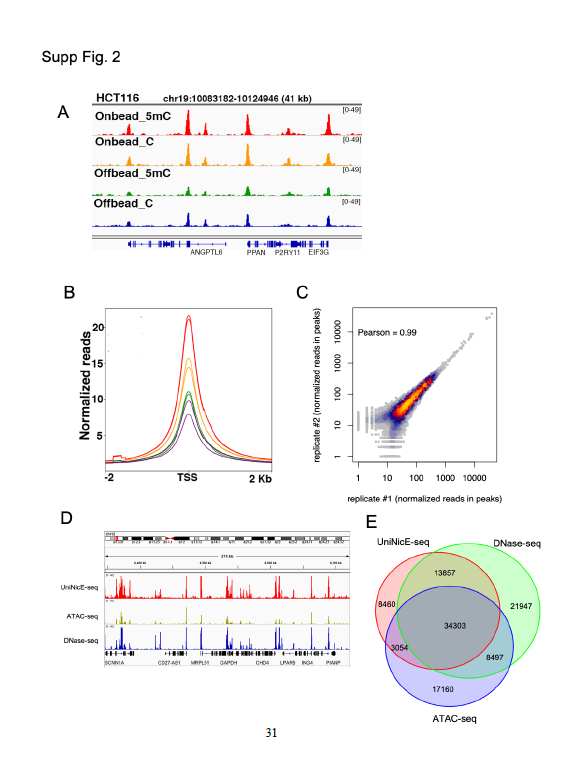

Supplement: Supplementary file 1 — Additional file 1: Supp Fig. 1 Optimization of universal NicE-seq (A) A schematic diagram of accessible chromatin labeling using dCTP or 5-mdCTP in the labeling reaction along with biotinylated-dCTP in the nucleotide mix. On-bead and off-bead represented presence of streptavidin magnetic beads for DNA capture and library preparation. (B) FRiP comparison between all 4 methods generated library that map to TSSs (+/-500 bp of TSS) and distal elements (>500 bp from TSS) from HCT116 cells. C and 5mC represents use of dCTP and 5-dCTP in the reaction mix. Supp Fig. 2: Optimization of accessible chromatin sequencing and comparison between UniNicE-seq, ATAC-seq and DNase-seq. (A) IGV screen shot of the normalized read density of the four NicE-seq conditions in HCT116 cells. (B) Distribution of the number of normalized HCT116 NicE-seq reads at transcription start sites (TSS) of human genes and the surrounding 2 Kb (- and +) regions. (C) Pearson correlation of normalized read densities in UniNicE-seq peaks of the 2 technical replicates in HCT116 demonstrating reproducibility. (D) IGV screen shot of the normalized read density of UniNicE-seq (top track), ATAC-seq (middle track) and DNase-seq (bottom track) in HCT116 (F) Overlap of HCT116 peaks called from 15 M unique alignments using UniNicE-seq, ATAC-seq and DNase-seq. Supp Fig. 3: Venn Diagram showing common and cell-type specific UniNicE-seq peaks between the three cell types. (A) HCT116, K562 and MCF7 accessible chromatin regions were analyzed. Peaks are called from 11 million random sampled deduplicated alignment pairs. Supp Fig. 4: UniNicE-seq of mouse T cells cells. (A) IGV screen shot of the normalized read density of the technical duplicates of UniNicE-seq libraries of HCT116 cells at different cell numbers. (B) Pairwise comparison between all Universal NicE-seq reads between different T cell numbers from 500, 5 and 25 K. Pearson’s correlation is indicated. Supp Fig. 5: Comparison between UniNicE-seq, ATAC-seq, Omini A [file 13148_2020_921_MOESM1_ESM.zip › Additional file Fig S2.PNG]

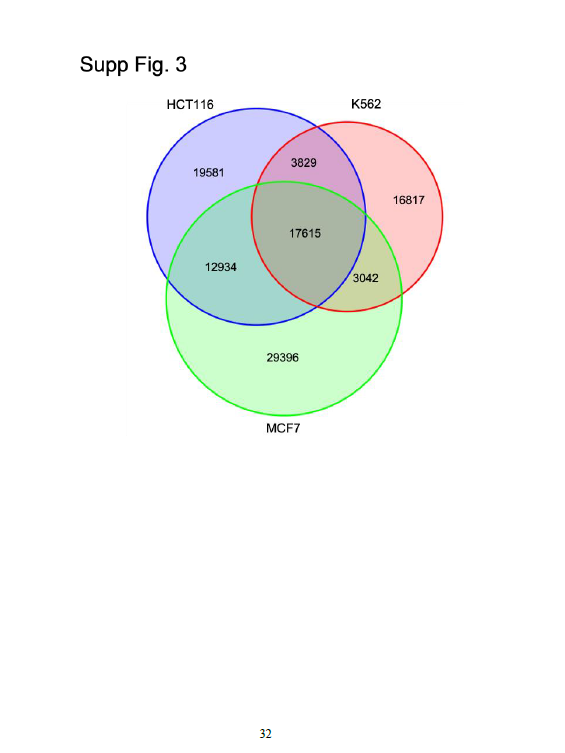

Supplement: Supplementary file 1 — Additional file 1: Supp Fig. 1 Optimization of universal NicE-seq (A) A schematic diagram of accessible chromatin labeling using dCTP or 5-mdCTP in the labeling reaction along with biotinylated-dCTP in the nucleotide mix. On-bead and off-bead represented presence of streptavidin magnetic beads for DNA capture and library preparation. (B) FRiP comparison between all 4 methods generated library that map to TSSs (+/-500 bp of TSS) and distal elements (>500 bp from TSS) from HCT116 cells. C and 5mC represents use of dCTP and 5-dCTP in the reaction mix. Supp Fig. 2: Optimization of accessible chromatin sequencing and comparison between UniNicE-seq, ATAC-seq and DNase-seq. (A) IGV screen shot of the normalized read density of the four NicE-seq conditions in HCT116 cells. (B) Distribution of the number of normalized HCT116 NicE-seq reads at transcription start sites (TSS) of human genes and the surrounding 2 Kb (- and +) regions. (C) Pearson correlation of normalized read densities in UniNicE-seq peaks of the 2 technical replicates in HCT116 demonstrating reproducibility. (D) IGV screen shot of the normalized read density of UniNicE-seq (top track), ATAC-seq (middle track) and DNase-seq (bottom track) in HCT116 (F) Overlap of HCT116 peaks called from 15 M unique alignments using UniNicE-seq, ATAC-seq and DNase-seq. Supp Fig. 3: Venn Diagram showing common and cell-type specific UniNicE-seq peaks between the three cell types. (A) HCT116, K562 and MCF7 accessible chromatin regions were analyzed. Peaks are called from 11 million random sampled deduplicated alignment pairs. Supp Fig. 4: UniNicE-seq of mouse T cells cells. (A) IGV screen shot of the normalized read density of the technical duplicates of UniNicE-seq libraries of HCT116 cells at different cell numbers. (B) Pairwise comparison between all Universal NicE-seq reads between different T cell numbers from 500, 5 and 25 K. Pearson’s correlation is indicated. Supp Fig. 5: Comparison between UniNicE-seq, ATAC-seq, Omini A [file 13148_2020_921_MOESM1_ESM.zip › Additional file Fig S3.PNG]

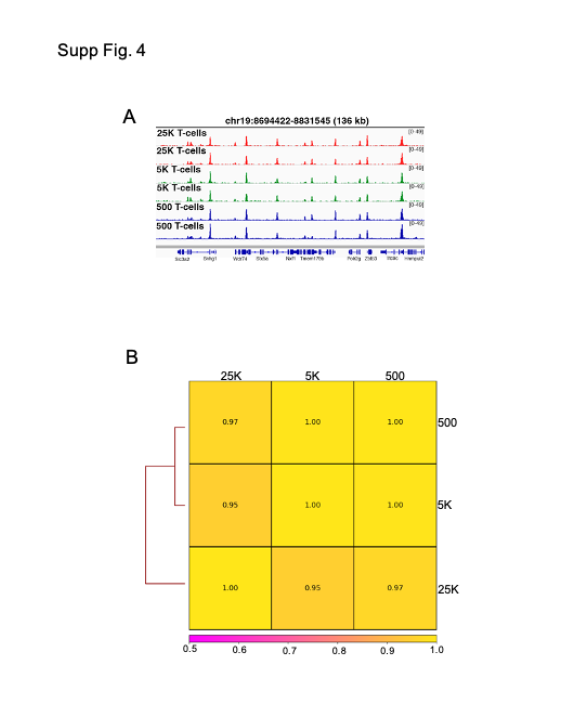

Supplement: Supplementary file 1 — Additional file 1: Supp Fig. 1 Optimization of universal NicE-seq (A) A schematic diagram of accessible chromatin labeling using dCTP or 5-mdCTP in the labeling reaction along with biotinylated-dCTP in the nucleotide mix. On-bead and off-bead represented presence of streptavidin magnetic beads for DNA capture and library preparation. (B) FRiP comparison between all 4 methods generated library that map to TSSs (+/-500 bp of TSS) and distal elements (>500 bp from TSS) from HCT116 cells. C and 5mC represents use of dCTP and 5-dCTP in the reaction mix. Supp Fig. 2: Optimization of accessible chromatin sequencing and comparison between UniNicE-seq, ATAC-seq and DNase-seq. (A) IGV screen shot of the normalized read density of the four NicE-seq conditions in HCT116 cells. (B) Distribution of the number of normalized HCT116 NicE-seq reads at transcription start sites (TSS) of human genes and the surrounding 2 Kb (- and +) regions. (C) Pearson correlation of normalized read densities in UniNicE-seq peaks of the 2 technical replicates in HCT116 demonstrating reproducibility. (D) IGV screen shot of the normalized read density of UniNicE-seq (top track), ATAC-seq (middle track) and DNase-seq (bottom track) in HCT116 (F) Overlap of HCT116 peaks called from 15 M unique alignments using UniNicE-seq, ATAC-seq and DNase-seq. Supp Fig. 3: Venn Diagram showing common and cell-type specific UniNicE-seq peaks between the three cell types. (A) HCT116, K562 and MCF7 accessible chromatin regions were analyzed. Peaks are called from 11 million random sampled deduplicated alignment pairs. Supp Fig. 4: UniNicE-seq of mouse T cells cells. (A) IGV screen shot of the normalized read density of the technical duplicates of UniNicE-seq libraries of HCT116 cells at different cell numbers. (B) Pairwise comparison between all Universal NicE-seq reads between different T cell numbers from 500, 5 and 25 K. Pearson’s correlation is indicated. Supp Fig. 5: Comparison between UniNicE-seq, ATAC-seq, Omini A [file 13148_2020_921_MOESM1_ESM.zip › Additional file Fig S4.PNG]

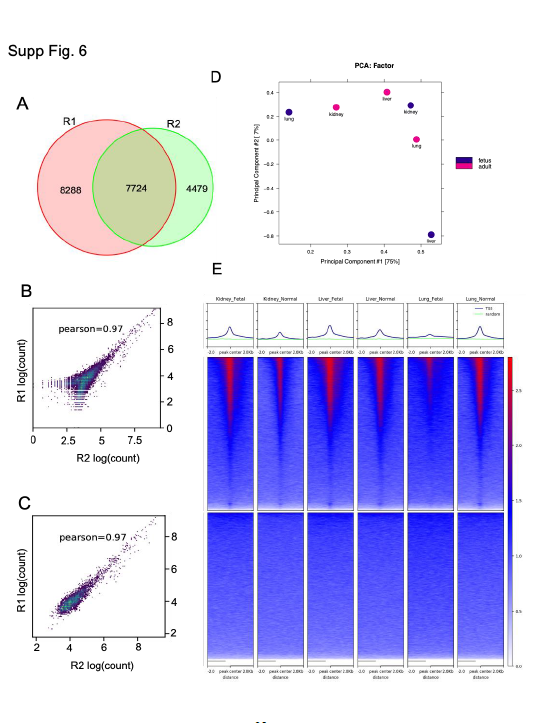

Supplement: Supplementary file 1 — Additional file 1: Supp Fig. 1 Optimization of universal NicE-seq (A) A schematic diagram of accessible chromatin labeling using dCTP or 5-mdCTP in the labeling reaction along with biotinylated-dCTP in the nucleotide mix. On-bead and off-bead represented presence of streptavidin magnetic beads for DNA capture and library preparation. (B) FRiP comparison between all 4 methods generated library that map to TSSs (+/-500 bp of TSS) and distal elements (>500 bp from TSS) from HCT116 cells. C and 5mC represents use of dCTP and 5-dCTP in the reaction mix. Supp Fig. 2: Optimization of accessible chromatin sequencing and comparison between UniNicE-seq, ATAC-seq and DNase-seq. (A) IGV screen shot of the normalized read density of the four NicE-seq conditions in HCT116 cells. (B) Distribution of the number of normalized HCT116 NicE-seq reads at transcription start sites (TSS) of human genes and the surrounding 2 Kb (- and +) regions. (C) Pearson correlation of normalized read densities in UniNicE-seq peaks of the 2 technical replicates in HCT116 demonstrating reproducibility. (D) IGV screen shot of the normalized read density of UniNicE-seq (top track), ATAC-seq (middle track) and DNase-seq (bottom track) in HCT116 (F) Overlap of HCT116 peaks called from 15 M unique alignments using UniNicE-seq, ATAC-seq and DNase-seq. Supp Fig. 3: Venn Diagram showing common and cell-type specific UniNicE-seq peaks between the three cell types. (A) HCT116, K562 and MCF7 accessible chromatin regions were analyzed. Peaks are called from 11 million random sampled deduplicated alignment pairs. Supp Fig. 4: UniNicE-seq of mouse T cells cells. (A) IGV screen shot of the normalized read density of the technical duplicates of UniNicE-seq libraries of HCT116 cells at different cell numbers. (B) Pairwise comparison between all Universal NicE-seq reads between different T cell numbers from 500, 5 and 25 K. Pearson’s correlation is indicated. Supp Fig. 5: Comparison between UniNicE-seq, ATAC-seq, Omini A [file 13148_2020_921_MOESM1_ESM.zip › Additional file Fig S6.PNG]

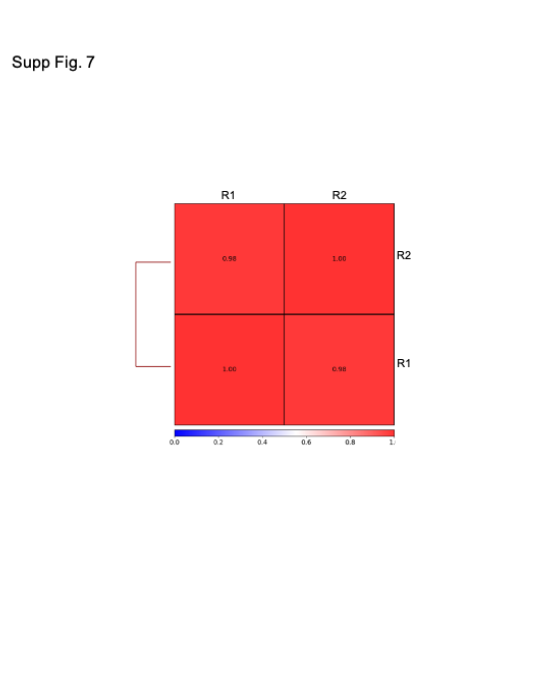

Supplement: Supplementary file 1 — Additional file 1: Supp Fig. 1 Optimization of universal NicE-seq (A) A schematic diagram of accessible chromatin labeling using dCTP or 5-mdCTP in the labeling reaction along with biotinylated-dCTP in the nucleotide mix. On-bead and off-bead represented presence of streptavidin magnetic beads for DNA capture and library preparation. (B) FRiP comparison between all 4 methods generated library that map to TSSs (+/-500 bp of TSS) and distal elements (>500 bp from TSS) from HCT116 cells. C and 5mC represents use of dCTP and 5-dCTP in the reaction mix. Supp Fig. 2: Optimization of accessible chromatin sequencing and comparison between UniNicE-seq, ATAC-seq and DNase-seq. (A) IGV screen shot of the normalized read density of the four NicE-seq conditions in HCT116 cells. (B) Distribution of the number of normalized HCT116 NicE-seq reads at transcription start sites (TSS) of human genes and the surrounding 2 Kb (- and +) regions. (C) Pearson correlation of normalized read densities in UniNicE-seq peaks of the 2 technical replicates in HCT116 demonstrating reproducibility. (D) IGV screen shot of the normalized read density of UniNicE-seq (top track), ATAC-seq (middle track) and DNase-seq (bottom track) in HCT116 (F) Overlap of HCT116 peaks called from 15 M unique alignments using UniNicE-seq, ATAC-seq and DNase-seq. Supp Fig. 3: Venn Diagram showing common and cell-type specific UniNicE-seq peaks between the three cell types. (A) HCT116, K562 and MCF7 accessible chromatin regions were analyzed. Peaks are called from 11 million random sampled deduplicated alignment pairs. Supp Fig. 4: UniNicE-seq of mouse T cells cells. (A) IGV screen shot of the normalized read density of the technical duplicates of UniNicE-seq libraries of HCT116 cells at different cell numbers. (B) Pairwise comparison between all Universal NicE-seq reads between different T cell numbers from 500, 5 and 25 K. Pearson’s correlation is indicated. Supp Fig. 5: Comparison between UniNicE-seq, ATAC-seq, Omini A [file 13148_2020_921_MOESM1_ESM.zip › Additional file Fig S7.PNG]

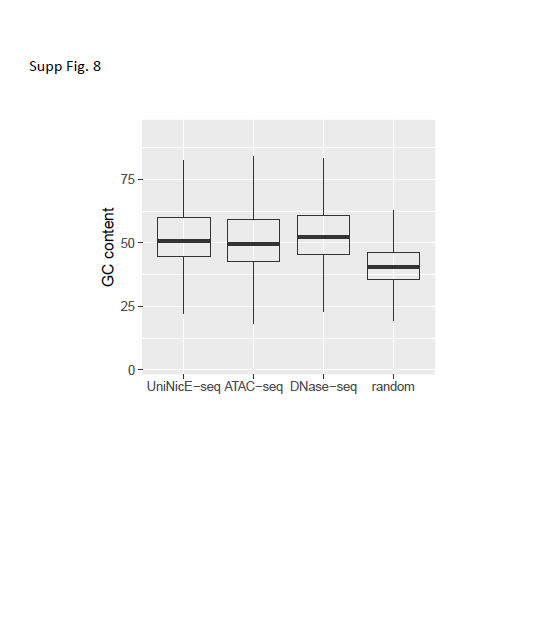

Supplement: Supplementary file 1 — Additional file 1: Supp Fig. 1 Optimization of universal NicE-seq (A) A schematic diagram of accessible chromatin labeling using dCTP or 5-mdCTP in the labeling reaction along with biotinylated-dCTP in the nucleotide mix. On-bead and off-bead represented presence of streptavidin magnetic beads for DNA capture and library preparation. (B) FRiP comparison between all 4 methods generated library that map to TSSs (+/-500 bp of TSS) and distal elements (>500 bp from TSS) from HCT116 cells. C and 5mC represents use of dCTP and 5-dCTP in the reaction mix. Supp Fig. 2: Optimization of accessible chromatin sequencing and comparison between UniNicE-seq, ATAC-seq and DNase-seq. (A) IGV screen shot of the normalized read density of the four NicE-seq conditions in HCT116 cells. (B) Distribution of the number of normalized HCT116 NicE-seq reads at transcription start sites (TSS) of human genes and the surrounding 2 Kb (- and +) regions. (C) Pearson correlation of normalized read densities in UniNicE-seq peaks of the 2 technical replicates in HCT116 demonstrating reproducibility. (D) IGV screen shot of the normalized read density of UniNicE-seq (top track), ATAC-seq (middle track) and DNase-seq (bottom track) in HCT116 (F) Overlap of HCT116 peaks called from 15 M unique alignments using UniNicE-seq, ATAC-seq and DNase-seq. Supp Fig. 3: Venn Diagram showing common and cell-type specific UniNicE-seq peaks between the three cell types. (A) HCT116, K562 and MCF7 accessible chromatin regions were analyzed. Peaks are called from 11 million random sampled deduplicated alignment pairs. Supp Fig. 4: UniNicE-seq of mouse T cells cells. (A) IGV screen shot of the normalized read density of the technical duplicates of UniNicE-seq libraries of HCT116 cells at different cell numbers. (B) Pairwise comparison between all Universal NicE-seq reads between different T cell numbers from 500, 5 and 25 K. Pearson’s correlation is indicated. Supp Fig. 5: Comparison between UniNicE-seq, ATAC-seq, Omini A [file 13148_2020_921_MOESM1_ESM.zip › Additional file Fig S8.PNG]

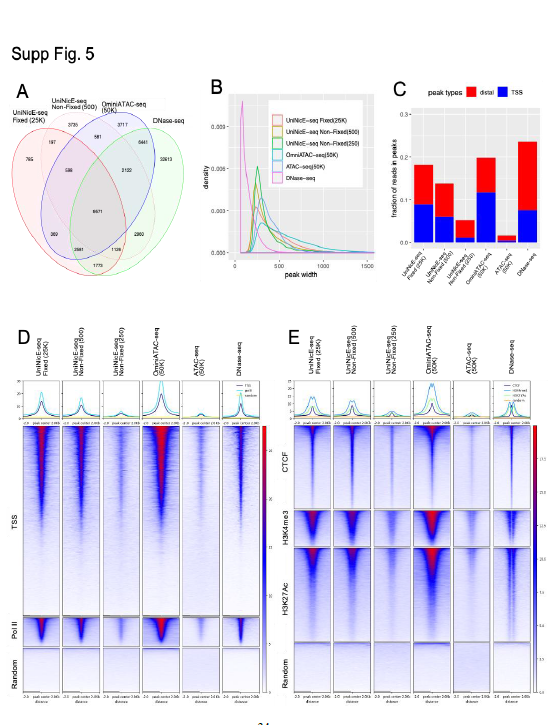

Supplement: Supplementary file 1 — Additional file 1: Supp Fig. 1 Optimization of universal NicE-seq (A) A schematic diagram of accessible chromatin labeling using dCTP or 5-mdCTP in the labeling reaction along with biotinylated-dCTP in the nucleotide mix. On-bead and off-bead represented presence of streptavidin magnetic beads for DNA capture and library preparation. (B) FRiP comparison between all 4 methods generated library that map to TSSs (+/-500 bp of TSS) and distal elements (>500 bp from TSS) from HCT116 cells. C and 5mC represents use of dCTP and 5-dCTP in the reaction mix. Supp Fig. 2: Optimization of accessible chromatin sequencing and comparison between UniNicE-seq, ATAC-seq and DNase-seq. (A) IGV screen shot of the normalized read density of the four NicE-seq conditions in HCT116 cells. (B) Distribution of the number of normalized HCT116 NicE-seq reads at transcription start sites (TSS) of human genes and the surrounding 2 Kb (- and +) regions. (C) Pearson correlation of normalized read densities in UniNicE-seq peaks of the 2 technical replicates in HCT116 demonstrating reproducibility. (D) IGV screen shot of the normalized read density of UniNicE-seq (top track), ATAC-seq (middle track) and DNase-seq (bottom track) in HCT116 (F) Overlap of HCT116 peaks called from 15 M unique alignments using UniNicE-seq, ATAC-seq and DNase-seq. Supp Fig. 3: Venn Diagram showing common and cell-type specific UniNicE-seq peaks between the three cell types. (A) HCT116, K562 and MCF7 accessible chromatin regions were analyzed. Peaks are called from 11 million random sampled deduplicated alignment pairs. Supp Fig. 4: UniNicE-seq of mouse T cells cells. (A) IGV screen shot of the normalized read density of the technical duplicates of UniNicE-seq libraries of HCT116 cells at different cell numbers. (B) Pairwise comparison between all Universal NicE-seq reads between different T cell numbers from 500, 5 and 25 K. Pearson’s correlation is indicated. Supp Fig. 5: Comparison between UniNicE-seq, ATAC-seq, Omini A [file 13148_2020_921_MOESM1_ESM.zip › Additional file Fig S5.PNG]

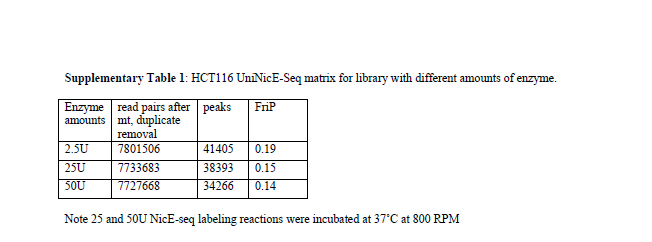

Supplement: Supplementary file 2 — Additional file 2: Supplementary Table 1: HCT116 UniNicE-Seq matrix for library with different amounts of enzyme. Note 25 and 50 U NicE-seq labeling reactions were incubated at 37 °C at 800 RPM. Supplementary Table 2: Quality control metrics of UniNicE-seq libraries applied to two human cell lines K562 and MCF7 in comparison to libraries made on and off beads with either with 5mdCTP or dCTP in the dNTP mix. We examined percentage of mitochondrial reads (“%mito”), number of total peaks and promoter peaks (+/- 500 bp of TSS) and enrichment of signal at TSSs (“FRiP (TSS peaks)”). Two technical replicates were conducted for each sample. All the values were calculated from a subsample of 11 million de-duplicated alignment pairs. Supplementary Table 3. Quality control metrics of UniNicE-seq libraries applied to mouse kidney tissues. 25 K fixed cells were compared with 25 K, 10 K, 1 K, 0.5 K and 0.25 K unfixed cells. Supplementary Table 4. a. Quality control metrics of UniNicE-seq libraries applied to human adult lung tissues. b. Quality control metrics of UniNicE-seq libraries applied to different human adult and fetal tissues.* *Here the replicates are merged together and downsized to 50 M aligned pairs for the downstream analysis. Supplementary Table 5. Quality control metrics of UniNicE-seq libraries applied to human FFPE liver tissue sections. Supplemental Table 6. External ChIP-seq data sets of various human and mouse tissue and cell types in this work. [file 13148_2020_921_MOESM2_ESM.zip › Additional file Table S1.PNG]

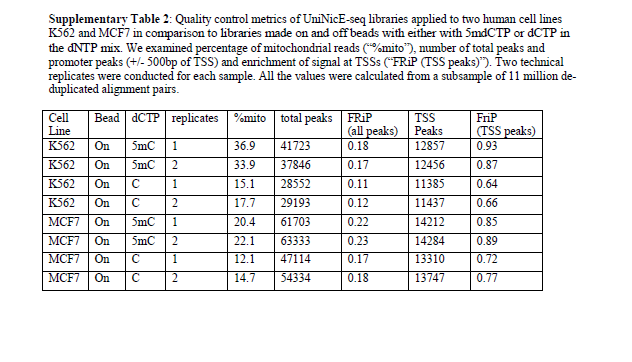

Supplement: Supplementary file 2 — Additional file 2: Supplementary Table 1: HCT116 UniNicE-Seq matrix for library with different amounts of enzyme. Note 25 and 50 U NicE-seq labeling reactions were incubated at 37 °C at 800 RPM. Supplementary Table 2: Quality control metrics of UniNicE-seq libraries applied to two human cell lines K562 and MCF7 in comparison to libraries made on and off beads with either with 5mdCTP or dCTP in the dNTP mix. We examined percentage of mitochondrial reads (“%mito”), number of total peaks and promoter peaks (+/- 500 bp of TSS) and enrichment of signal at TSSs (“FRiP (TSS peaks)”). Two technical replicates were conducted for each sample. All the values were calculated from a subsample of 11 million de-duplicated alignment pairs. Supplementary Table 3. Quality control metrics of UniNicE-seq libraries applied to mouse kidney tissues. 25 K fixed cells were compared with 25 K, 10 K, 1 K, 0.5 K and 0.25 K unfixed cells. Supplementary Table 4. a. Quality control metrics of UniNicE-seq libraries applied to human adult lung tissues. b. Quality control metrics of UniNicE-seq libraries applied to different human adult and fetal tissues.* *Here the replicates are merged together and downsized to 50 M aligned pairs for the downstream analysis. Supplementary Table 5. Quality control metrics of UniNicE-seq libraries applied to human FFPE liver tissue sections. Supplemental Table 6. External ChIP-seq data sets of various human and mouse tissue and cell types in this work. [file 13148_2020_921_MOESM2_ESM.zip › Additional file Table S2.PNG]

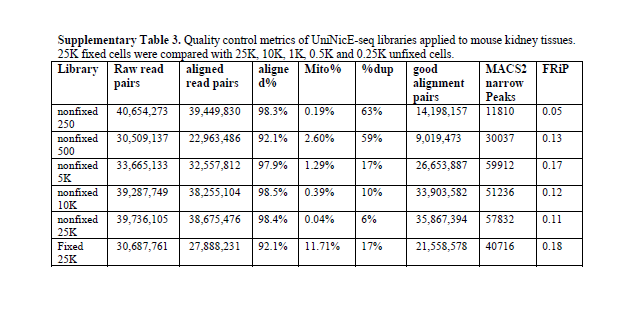

Supplement: Supplementary file 2 — Additional file 2: Supplementary Table 1: HCT116 UniNicE-Seq matrix for library with different amounts of enzyme. Note 25 and 50 U NicE-seq labeling reactions were incubated at 37 °C at 800 RPM. Supplementary Table 2: Quality control metrics of UniNicE-seq libraries applied to two human cell lines K562 and MCF7 in comparison to libraries made on and off beads with either with 5mdCTP or dCTP in the dNTP mix. We examined percentage of mitochondrial reads (“%mito”), number of total peaks and promoter peaks (+/- 500 bp of TSS) and enrichment of signal at TSSs (“FRiP (TSS peaks)”). Two technical replicates were conducted for each sample. All the values were calculated from a subsample of 11 million de-duplicated alignment pairs. Supplementary Table 3. Quality control metrics of UniNicE-seq libraries applied to mouse kidney tissues. 25 K fixed cells were compared with 25 K, 10 K, 1 K, 0.5 K and 0.25 K unfixed cells. Supplementary Table 4. a. Quality control metrics of UniNicE-seq libraries applied to human adult lung tissues. b. Quality control metrics of UniNicE-seq libraries applied to different human adult and fetal tissues.* *Here the replicates are merged together and downsized to 50 M aligned pairs for the downstream analysis. Supplementary Table 5. Quality control metrics of UniNicE-seq libraries applied to human FFPE liver tissue sections. Supplemental Table 6. External ChIP-seq data sets of various human and mouse tissue and cell types in this work. [file 13148_2020_921_MOESM2_ESM.zip › Additional file Table S3.PNG]

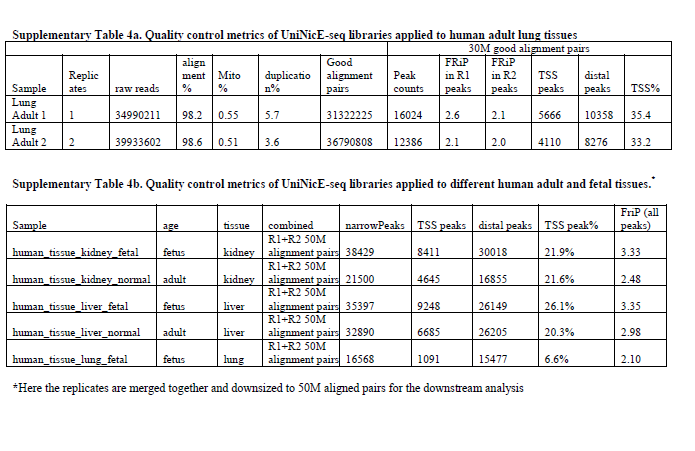

Supplement: Supplementary file 2 — Additional file 2: Supplementary Table 1: HCT116 UniNicE-Seq matrix for library with different amounts of enzyme. Note 25 and 50 U NicE-seq labeling reactions were incubated at 37 °C at 800 RPM. Supplementary Table 2: Quality control metrics of UniNicE-seq libraries applied to two human cell lines K562 and MCF7 in comparison to libraries made on and off beads with either with 5mdCTP or dCTP in the dNTP mix. We examined percentage of mitochondrial reads (“%mito”), number of total peaks and promoter peaks (+/- 500 bp of TSS) and enrichment of signal at TSSs (“FRiP (TSS peaks)”). Two technical replicates were conducted for each sample. All the values were calculated from a subsample of 11 million de-duplicated alignment pairs. Supplementary Table 3. Quality control metrics of UniNicE-seq libraries applied to mouse kidney tissues. 25 K fixed cells were compared with 25 K, 10 K, 1 K, 0.5 K and 0.25 K unfixed cells. Supplementary Table 4. a. Quality control metrics of UniNicE-seq libraries applied to human adult lung tissues. b. Quality control metrics of UniNicE-seq libraries applied to different human adult and fetal tissues.* *Here the replicates are merged together and downsized to 50 M aligned pairs for the downstream analysis. Supplementary Table 5. Quality control metrics of UniNicE-seq libraries applied to human FFPE liver tissue sections. Supplemental Table 6. External ChIP-seq data sets of various human and mouse tissue and cell types in this work. [file 13148_2020_921_MOESM2_ESM.zip › Additional file Table S4.PNG]

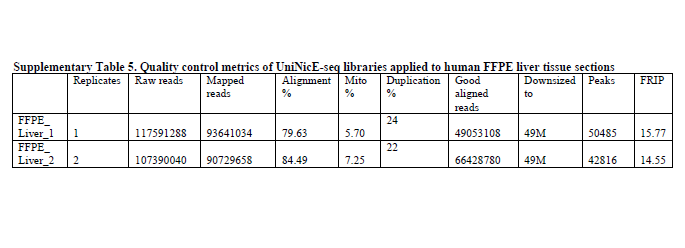

Supplement: Supplementary file 2 — Additional file 2: Supplementary Table 1: HCT116 UniNicE-Seq matrix for library with different amounts of enzyme. Note 25 and 50 U NicE-seq labeling reactions were incubated at 37 °C at 800 RPM. Supplementary Table 2: Quality control metrics of UniNicE-seq libraries applied to two human cell lines K562 and MCF7 in comparison to libraries made on and off beads with either with 5mdCTP or dCTP in the dNTP mix. We examined percentage of mitochondrial reads (“%mito”), number of total peaks and promoter peaks (+/- 500 bp of TSS) and enrichment of signal at TSSs (“FRiP (TSS peaks)”). Two technical replicates were conducted for each sample. All the values were calculated from a subsample of 11 million de-duplicated alignment pairs. Supplementary Table 3. Quality control metrics of UniNicE-seq libraries applied to mouse kidney tissues. 25 K fixed cells were compared with 25 K, 10 K, 1 K, 0.5 K and 0.25 K unfixed cells. Supplementary Table 4. a. Quality control metrics of UniNicE-seq libraries applied to human adult lung tissues. b. Quality control metrics of UniNicE-seq libraries applied to different human adult and fetal tissues.* *Here the replicates are merged together and downsized to 50 M aligned pairs for the downstream analysis. Supplementary Table 5. Quality control metrics of UniNicE-seq libraries applied to human FFPE liver tissue sections. Supplemental Table 6. External ChIP-seq data sets of various human and mouse tissue and cell types in this work. [file 13148_2020_921_MOESM2_ESM.zip › Additional file Table S5.PNG]

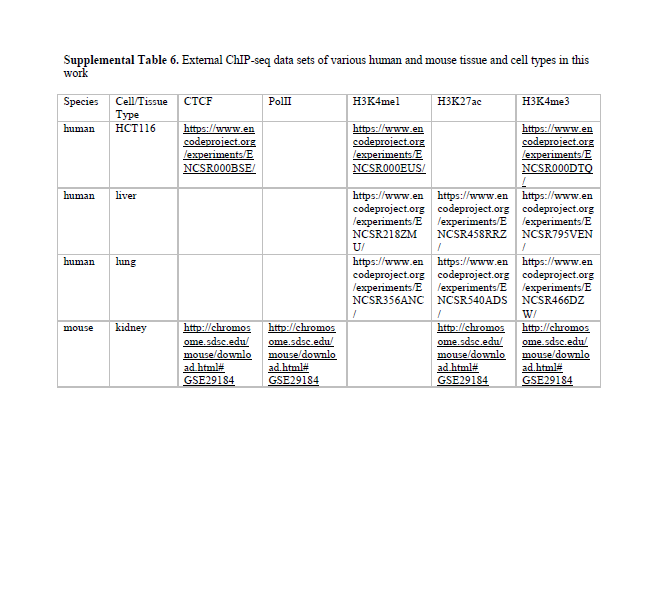

Supplement: Supplementary file 2 — Additional file 2: Supplementary Table 1: HCT116 UniNicE-Seq matrix for library with different amounts of enzyme. Note 25 and 50 U NicE-seq labeling reactions were incubated at 37 °C at 800 RPM. Supplementary Table 2: Quality control metrics of UniNicE-seq libraries applied to two human cell lines K562 and MCF7 in comparison to libraries made on and off beads with either with 5mdCTP or dCTP in the dNTP mix. We examined percentage of mitochondrial reads (“%mito”), number of total peaks and promoter peaks (+/- 500 bp of TSS) and enrichment of signal at TSSs (“FRiP (TSS peaks)”). Two technical replicates were conducted for each sample. All the values were calculated from a subsample of 11 million de-duplicated alignment pairs. Supplementary Table 3. Quality control metrics of UniNicE-seq libraries applied to mouse kidney tissues. 25 K fixed cells were compared with 25 K, 10 K, 1 K, 0.5 K and 0.25 K unfixed cells. Supplementary Table 4. a. Quality control metrics of UniNicE-seq libraries applied to human adult lung tissues. b. Quality control metrics of UniNicE-seq libraries applied to different human adult and fetal tissues.* *Here the replicates are merged together and downsized to 50 M aligned pairs for the downstream analysis. Supplementary Table 5. Quality control metrics of UniNicE-seq libraries applied to human FFPE liver tissue sections. Supplemental Table 6. External ChIP-seq data sets of various human and mouse tissue and cell types in this work. [file 13148_2020_921_MOESM2_ESM.zip › Additional file Table S6.PNG]
