# Supplementary material for: Universal NicE-seq for high-resolution accessible chromatin profiling for formaldehyde-fixed and FFPE tissues
Source: Clin Epigenetics. 2020 Sep 22;12:143. doi: 10.1186/s13148-020-00921-6 (PMC7507628; doi:10.1186/s13148-020-00921-6)
Supplement: Supplementary file 3 — Additional file 3: Supplementary text 1. Detailed stepwise protocol: universal NicE-seq (25000-250000 HCT116 cells). [file 13148_2020_921_MOESM3_ESM.pdf]

Supplementary text 1

DETAILED PROTOCOL:
UNIVERSAL NICE-SEQ (25000-250000 HCT116 CELLS)

1. CROSSLINKING AND HARVESTING CELLS

FORMALDEHYDE must be handled with GLOVES and in a FUMEHOOD

MATERIALS

- For HCT116 cells: Culture in McCoys 5A medium (ThermoFisher Scientific #16600082) supplemented with 10% Fetal Bovine Serum (GemCell #100-500).
- TrypLE (ThermoFisher Scientific #12605028, RT before use) (trypsin will release cells from bottom of falcon dish)
- 50 mL tube and large pipette tips + automatic pipet
- cell culture flask
- Trypan Blue Stain (0.4%)
- Hemacytometer and microscope
- 1.5mL eppendorf for cell harvest; 1.5 ml DNA lobind tube (Eppendorf AG #022431021)
- 16 % formaldehyde (Thermo Scientific#28908)
- 1x PBS (Gibco#70011-044)
- 2.5 M Glycine (Sigma # G7126)
- End-over end bench top rotation wheel (VWR)

LAMINAR FLOW HOOD; NEVER SPIN CELLS AT HIGHER SPEED THAN 1500Xg!

Cell culture and harvesting cells:

- Take cells from the incubator and visually check their health under the microscope.
- Remove old medium in the flask and transfer 50 ml to a sterile conical centrifuge tube.
  - Add **5-10 mL TrypLE** to the adherent cells in the flask, and incubate **5 min @ 37 °C**.
  - Gently tap to detach cells, pipet cells to the old medium containing sterile conical centrifuge tube (the old medium/serum will inhibit the activity of the trypsin)
  - Spin down **5 min at room temp, 1500 rpm**, remove supernatant (decant)
  - **Add 5mL medium** (McCoys) to 50 mL tube, resuspend gently by pipetting.
  - Optional: sub-culture of cells
  - Rest of the cells is now harvested for universal NicE-seq.
    - Count cells: Dilute small amounts of cells (**1:1**) with **Trypan Blue Stain** (gives you a dilution factor of 2) (eg., take 100μL cells and 100μL stain). Pipet into small edge of the hemacytometer ie counting chamber.
    - Count the number of cells per squares (16) in upper and lower counting chambers. Presence of blue cells indicate dead cells, don't count these.
    - Calculate the number of cells per mL:

        - Average number of cells X dilution factor (2) / volume of the counting chamber (0.0001mL)
    - Calculate how many cells in total. Take 10^6 cells and transfer to 1.5 ml Eppendorf tube. Spin down **(5 min @ RT, 1500 rpm**). Remove supernatant liquid.
    - Wash in **1 mL 1x PBS** (resuspend gently), spin down (**5 min, RT at 1500 rpm**). Remove supernatant liquid.

- X-linking: Add **937.5 µL 1x PBS** and transfer cells to 1.5 mL Eppendorf tube. Add **62.5 µL 16 % formaldehyde** (final concentration = 1% formaldehyde). Incubate cells **10 min @ RT** by end-over end rotation (rotation wheel).
- Quench reaction by **adding 125 mM Glycine** (for a volume of 1mL add **52 µL** of the 2.5 M stock): Incubate **@5 min. RT**, end-over end rotation.
- Wash cells **twice with 1mL 1x PBS**. (Spin down **1 min @ 1500 RPM, RT**; Remove supernatant and cells may be stored at -80°C.
    - Resuspend in **1x PBS** (0.5 ml) and count the number of cells and make aliquots depending on how many cells will be needed for downstream work (ideally 400 µls for 1 million cells, that will give 250,000 in 0.1 ml).
- Note: Cells may be lost during centrifugation steps therefore the above counting step is crucial for universal NicE-seq.

2. ACCESSIBLE CHROMATIN LABELLING (DAY 1):

MATERIALS

- Prepare Cytosolic Buffer (storage temp 4˚C):

Final concentration	Stock Solution
15mM	Tris-HCl pH 7,5
5mM	$MgCl_2$
60mM	KCl
0.5mM	DTT
15mM	NaCl
300mM	Sucrose
1%	NP40
	MQ

- Centrifuge at 4°C
- 1x PBS
- Prepare Accessible Chromatin Labelling buffer (for one sample reaction)

Volume	Enzyme and dNTPs	Final conc
20μL	Buffer: NEB2 (10x)	2x
1μL	DNA polymerase I (10U)	10U
5μL	Nt.CviPII enzyme 2U/ μl (**make a 1:10 dilution of the** 2U **stock in 1xPBS**)	1U
3μL	Biotin-dNTP mixture	
71 μL	Nuclease free water	

- Biotin-labeled dNTP mixture 10X:

Final concentration (10X)	Stock Solution
240 μM	dATP
240 μM	dmCTP
300 μM	dGTP
300 μM	dTTP
60 μM	Biotin-14-dATP
60 μM	Biotin-14-dCTP

MATERIALS

- 37°C Incubator
- 0.5 M EDTA (Invitrogen #15575-038)
- RNase A (Invitrogen #12091021)
- Proteinase K (NEB, P8107S)
- 20% SDS (TEKNOVA #S0295)
- 65°C heatblock
- Centrifuge at room temp
- Phenol:Chloroform: Isoamyl Alcohol (Invitrogen#15593-031)
- Isopropanol (Pharmco-Aaper#231HPLC99)
- Glycogen (Sigma#10901393001)
- 80% EtOH in nuclease free water
- 1x TE buffer

- Start with 25-250.000 crosslinked cells suspended in **1x PBS** (25µL).
- Add 300 µL **Cytosolic Buffer** to the cells and incubate for **10 minutes on ice** with occasional mixing.
  - Nuclei can be visualized under the microscope at this point (Circular with smooth edges).
- Spin the nuclei down for **10 minutes @ 1000 g (or 3000 rpm), 4 °C**. Discard the supernatant.
  - To make sure you don't lose the nuclei leave a small amount (10-20µL) of supernatant in the tube.
- Add **100 µL cold 1x PBS buffer** and tap/pipet gently to dissolve the cells.
- Add **100 µL of Accessible Chromatin labeling buffer (at this point total volume is 200 µL)**. Incubate for **2h @ 37°C**.
- Add **20µL 0.5M EDTA** and **2µL RNaseA** to each sample. Incubate **30 minutes @ 37 °C** to digest RNA.
- Reverse Crosslink sample by adding **20 µL proteinase K** and **20 µL 20% SDS**. Incubate **ON @ 65 °C**.

3. DNA EXTRACTION (DAY 2):

- Extract Biotin labeled genomic DNA via phenol/chloroform extraction:
  - Add **250µL phenol/chloroform**. Vertex 3X times, 5-10 sec each.
  - Centrifuge **10 minutes at 14000 rpm, 4 °C**. Transfer aqueous phase (upper part) to new Eppendorf tube (will be maximum 260 µL but if starting from a lot of cells you don't need to take so much as this will increase the chances of collecting the interphase that contains proteins).
  - Add 0**.7 V isopropanol** and **2 µL glycogen**. Incubate for **2h @ -80 °C** or **ON @ -20 °C**.
  - Centrifuge **10 min @ 14000 rpm, 4 °C**.
  - Carefully wash pellet (not resuspending!) **with 500 µL 80 % EtOH.** Centrifuge **10 min @ 14000 rpm, 4°C.** (So just add EtOH and centrifuge, take of EtOH and air dry)
  - Air dry sample and resuspend DNA in **50 µL 1x TE buffer.**
- Measure DNA concentration by Qubit High Sensitivity method (in case concentration too high: dilute 10x; PS: when starting with high amounts of cells (~250, 000) concentrations will be high so making a dilution is standard procedure.

- Extract Biotin labeled genomic DNA by column purification using Monarch genomic DNA purification kit (NEB cat no: T3010)
- Measure DNA concentration by Qubit High Sensitivity method (in case concentration too high: dilute 10x; PS: when starting with high amounts of cells (~250, 000) concentrations will be high so making a dilution is standard procedure.

QUALITY CONTROL (Optional):

Open Chromatin Labeling efficiency can be analyzed via dot blot on genomic DNA.

MATERIALS

- Thermoblock @ 95°C
- Ice-water bath
- Positively charged nylon membranes (Roche, 11209299001)
- UV light
- Blotting-grade blocker (Bio-Rad #170-6404)
- 1x PBS with 0.1% Tween20
- goat anti-biotin-HRP antibody (CST, #7075)
- LumiGLO reagent (CST, #7003)

- Denature gDNA by heating for **3 min @ 95°C**, incubate **3 min** in an ice-water bath
- Make a serial dilution (1 µg; 0.5 µg; 0.25 µg; 0.125 µg) of gDNA in MQ on ice. Never exceed a total volume of 5 µL per spot.
- Prepare a positively charged nylon membrane by marking with a pencil the circles where you will spot the DNA. Spot the dilution series on the membrane and let dry.
- Cut mark the upper left corner (for orientation).
- Wet the membrane by dripping MQ on top of it so that it is fully hydrated.
- Cross-link by UV: put box with membrane in machine. Power → optimal crosslink → start. repeat once more.
- Wash membrane wit **1x PBS-T**. Transfer the membrane to a square protein gel box (with lid).
- Block the membrane with **5% skimmed milk in 1x PBS-T** for **0.5 to 1h @ RT** on a shaker.
- Add **1:2000 anti-biotin-HRP (5µL)** to the milk on the membrane to probe the DNA. Incubate **1h @ RT** on a shaker.
- Detect biotin signal with LumiGLO reagent.

4. UNINICE-SEQ LIBRARY CONSTRUCTION

MATERIALS

- Covaris S2 sonicator
- Covaris microtubes (Covaris #500330)
- NEB Ultra II DNA library prep kit for Illumina (NEB #E7645)
- PCR machine
- Prepare **2x High Salt Buffer**

Final concentration	Product
10mM	Tris-HCl pH 8
2M	NaCl
1mM	EDTA
	MQ

- Prepare **1x High Salt Buffer with 0.05% Triton-X100**

Final concentration	Product	Stock	For 10 mL
1x	High Salt Buffer	2x	5 mL
0.05%	TritonX 100	10 %	50 µL
	MQ		4,950 mL

- DNA LoBind Eppendorf tubes
- Gelatin from cold water fish skin (Sigma #G7765)
- Magnetic Streptavidin beads (ThermoFisher Scientific #650.02)
- Blocked Streptavidin beads (Blocked ON in 0.1% Fish Gelatin in 1xPBS)
- End-over end rotator
- Nuclease free water
- Index primers (different index number per sample)
- AMPure XP beads (Beckman Coulter#A63881)
- 0.1x TE buffer
- Qubit
- Bioanalyzer

- Take 200 ng of DNA and sonicate:
  - Transfer to Covaris microtube and add **up to 50 µL 1x TE buffer**
  - Sonicate using the following settings to obtain 150 bp fragments. To do this, insert the tube into the holder, and simply select "Open" and select the program named "Covaris 200 for 50µL" on the computer. Click "start".
    1. Intensity: 5
    2. Duty Cycle: 10 %
    3. Cycles per burst: 200
    4. Treatment time: 2 min

- Prepare library: (Don't vortex the samples!)
  - **DNA pull-down:**
  - Transfer the Transfer sonicated DNA to PCR tube
  - For low number of cells add **15 µL** of Blocked Streptavidin beads. For high # of cells add **30 µL** of beads.
  - Add **1 mL of 1x High Salt Buffer** (CAUTION: The buffer described above is 2x! So **add 500 µL of 1x Buffer and 500 µL MQ**)
  - Incubate for **2hrs @ 4 °C** on end-over end rotator.
  - Put on magnetic rack. When solution is clear: Remove and wash beads for **5 minutes** with **1mL cold High Salt Buffer containing 0.05% TritonX 100**.
  - Repeat wash steps 3 times more (4 times in total)
  - Wash beads once with **1 mL 1x TE buffer** by inverting a couple of times (1x TE can be **@ RT**).
  - Resuspend beads in **50 µL 1x TE buffer. These beads will be used for the library preparation till PCR amplification step. Therefore, you should be careful not to lose beads.**
  - **End-repair/dA tailing:** Combine the following per sample
    1. **50 µL** of fragmented DNA
    2. **3 µL of NEB Next Ultra II End Prep Enzyme Mix**
    3. **7 µL of NEB Next Ultra II End Prep Reaction Buffer**

    Mix well and incubate at 20 °C for 30 min and at 65 °C for 30 min (Use a PCR machine Sometimes, you can tap the tube during reaction.)
  - **Washing:**

1. Wash the bead with High Salt Buffer with 0.05% Triton and incubate it on end-over end rotator for 5 min at R/T
2. Repeat twice more
3. Add 1 mL of 1X TE and incubate on end-over end rotator for 5 min at R/T
4. After remove the solution, re-suspend beads in **60 µL 1x TE buffer**

- **Adaptor Ligation:**
  1. 60 µL of End Prep Reaction Mixture
  2. **30 µL of NEB Next Ultra II ligation Master Mix**
  3. **1 µL of NEB Next ligation Enhancer**
  4. **1 µL of 1:10 diluted** (recommended for low amounts of DNA) **NEB Next Adaptor for Illumina**

  Incubate **2h-ON @ RT**.
- Wash the bead with High Salt Buffer with 0.05% Triton and incubate it on end-over end rotator for 5 min at R/T
- Repeat
- Add 1 mL of 1X TE and incubate on end-over end rotator for 5 min at room temp
- After remove the solution, re-suspend beads in **16 µL 1x TE buffer**
- Add 3 µl USER enzyme (NEB #M5508) and incubate at 37˚C for 20 min

- PCR amplification. Depending on the amount of DNA you can modify the PCR conditions.
  - Mix:
    1. **19 µL** of adaptor ligated DNA fragments that are bound to the beads and treated with USER
    2. **3 µL Index primer** (10 µM)
    3. **3 µL Universal primer** (10 µM)
    4. **25 µL NEB Ultra II Q5 Master Mix**
    5. Total volume of 50 µL of the final reaction.
  - Set up PCR:
    1. 30 sec @ 98 °C; initial denaturation
    2. 10 sec @ 98 °C; denaturation
    3. 30 sec @ 65 °C; annealing
    4. 45 sec @ 65 °C; extension

       (steps 2–4: 8 cycles)
    5. 5 min @ 72 °C; final extension
    6. hold @ 4 °C
  - Note: Depending on the amount of DNA, you can modify the PCR conditions. If your DNA is below 50 ng, you can increase the PCR cycles up to 12 cycles. In general, I don't use more than 12 cycles because it may cause amplify non-specific DNA. In case, if your DNA is very low such as below 50 ng, you can use all beads as template DNA for PCR
  - **<u>Put AMPure beads at RT after Set up PCR</u>**

- Clean up PCR using AMPure beads.
  - Set PCR reaction on magnetic rack. Transfer solution to new DNA LoBind tube and add **0.9 V** (so here **45 µL**) AMPure beads at room temp

- Incubate **10-15 min @ RT**
- Put samples on magnetic rack. When the solution looks clear, remove it and wash the beads twice with **200 µL 80 % EtOH** by slowly pipetting the EtOH on the beads WITHOUT REMOVING THE EPPENDORFS OFF OF THE RACK OR RESUSPENDING THE BEADS!! It is important that the beads do not move, or you could lose the DNA that is bound to them!! So just pipet the EtOH, and wait 30-60 seconds, remove it from the beads and repeat. After removing the EtOH for a second time, quickly spin down (short spin) the tubes to capture the remaining EtOH at the bottom of the tube and remove it. It is also reccomnded that one should use freshly prepared 80% ethanol for AMPure beads.
- When all EtOH is removed, resuspend the beads in **10 µL 0.1x TE buffer (**don't let the beads dry**).**
- Put back on the magnetic rack and transfer the solution to a new tube to get rid of the beads.
- Measure the amount of DNA using the Qubit hsDNA protocol. If the concentration is > 1 ng/µL, the library prep went well and it will be acceptable level for the Next-seq requirement.
- Analyze the DNA on the Bioanalyzer to check the actual library quantity.

Appendix 1: Making universal NicE-seq library from low cell numbers (250-5K)

- Start with crosslinked cells as per universal NicE-seq
- Major change at adaptor ligation: dilute adaptor 1:30 and use 1 µl for ligation
- PCR cycle may be increased up to 12
- If adaptor dimers are visible at bioanalyzer assay, re-purify the library with AMpure beads (Beckman Coulter)

Appendix 2: NicE-view (Nicking enzyme assisted viewing) of accessible chromatin

MATERIALS

- Prepare Cytosolic Buffer (storage temp 4˚C) as described for universal NicE-seq
- Centrifuge at 4°C
- 1x PBS
- Prepare Accessible Chromatin Labelling solution (for one sample reaction)

	Enzyme and dNTPs	Volume
	Buffer: NEB2 (10x)	100µL
	DNA polymerase I (50U)	5µL
	Nt.CviPII enzyme 2U/ µl (**make a 1:10 dilution of the 2U stock in 1xPBS**)	1.5µL
	labeled-dNTP mixture	10µL
	Nuclease free water	883.5 µL

- This case fluorescein/TexasRed-labeled dNTP mixture 10X:

Final concentration (10X)	Stock Solution
240 µM	dATP
300 µM	dmCTP
300 µM	dGTP
300 µM	dTTP
60 µM	Fluorescein-12-dATP (Perkin Elmer, NEL465001EA)/ 6 µM of TexasRed-5-dATP (Perkin Elmer, NEL471001EA)

- Start with crosslinked cells on slide.
- Add 1000 µL **Cytosolic Buffer** to the cells and incubate for **10 minutes on ice/cold 4˚C**.
- Wash once with cold 1X PBS
- Add 0.5-1 ml of Accessible Chromatin Labelling solution and incubate at 37°C for 2 h in a humidified chamber
- Add 80 µL of 0.5 M EDTA and 2 µg of RNase A are added to the labeling reaction and incubated at 37°C for 30 min
- Wash once the slides with 1XPBS at 55°C for 15 min to remove fluorescent background
- 3x washes with PBS for 5 min at RT
- Dry slides and mount using Prolong Gold antifade reagent with DAPI (Invitrogen, P36935)
- Ready for microscopy

Appendix 3: NicE-viewSeq (Nicking enzyme assisted viewing and sequencing) of accessible chromatin

MATERIALS

- Prepare Cytosolic Buffer (storage temp 4˚C) as described for universal NicE-seq
- Centrifuge at 4°C
- 1x PBS
- Prepare Accessible Chromatin Labelling solution (for one sample reaction)
- cell lysis buffer 50 mM Tris-Cl, pH 7.5, 1 mM EDTA, 1% SDS and 0.2 M NaCl.

	Enzyme and dNTPs	Volume
	Buffer: NEB2 (10x)	100µL
	DNA polymerase I (50U)	5µL
	Nt.CviPII enzyme 2U/ µl (**make a 1:10 dilution of the** 2U **stock in 1xPBS**)	1.5µL
	labeled-dNTP mixture	10µL
	Nuclease free water	883.5 µL

- This case fluorescein/TexasRed-labeled dNTP mixture 10X:

Final concentration (10X)	Stock Solution
240 µM	dATP
240 µM	dmCTP
300 µM	dGTP
300 µM	dTTP
60 µM	Fluorescein-12-dATP (Perkin Elmer, NEL465001EA)/
60 µM	Biotin-14-dCTP

- Start with crosslinked cells on slide.
- Add 1000 µL **Cytosolic Buffer** to the cells and incubate for **10 minutes on ice/cold 4˚C**.
- Wash once with cold 1X PBS
- Add 1 ml of Accessible Chromatin Labelling solution and incubate at 37°C for 2 h in a humidified chamber
- Add 80 µL of 0.5 M EDTA and 2 µg of RNase A are added to the labeling reaction and incubated at 37°C for 30 min
- Wash the slides with 1XPBS at 55°C for 15 min to remove fluorescent background
- Visual assay under microscope if needed
- Lysed cells on slide at room temperature with cell lysis buffer
- Carefully transfer to 1.5 ml Eppendorf tube and add 2 ml of proteinase K (NEB, P8107S), incubation at 65°C O/N
- Purify DNA and make library as per Universal NicE-seq protocol.

Appendix 4: One tube NicE-FFPE-Seq (Nicking enzyme assisted sequencing of FFPE) of 5-10 µm tissue section

- Prepare Cytosolic Buffer (storage temp 4˚C):

Final concentration	Stock Solution
15mM	Tris-HCl pH 7,5
5mM	$MgCl_2$
60mM	KCl
0.5mM	DTT
15mM	NaCl
300mM	Sucrose
1%	NP40
	MQ

- Centrifuge at 4°C
- 1x PBS
- Prepare Accessible Chromatin Labelling buffer (for one sample reaction)

Volume	Enzyme and dNTPs	Final conc
20µL	Buffer: NEB2 (10x)	2x
1µL	DNA polymerase I (10U)	10U
5µL	Nt.CviPII enzyme 2U/ µl (**make a 1:10 dilution of the** 2U **stock in 1xPBS**)	1U
3µL	Biotin-dNTP mixture	
71 µL	Nuclease free water	

- Biotin-labeled dNTP mixture 10X:

Final concentration (10X)	Stock Solution
240 µM	dATP
240 µM	dmCTP
300 µM	dGTP
300 µM	dTTP
60 µM	Biotin-14-dATP
60 µM	Biotin-14-dCTP

- Start with 5-10 µm FFPE section on slide.
- Add 500 µL **Mineral oil (Sigma)** to the slide and incubate for **20 minutes at 52˚C**.
- Transfer the slide to coupling jars/plate with 100% EtOH 5 min RT/ 100% EtOH 5 min RT/90% EtOH 5 min RT/100% EtOH 5 min RT/80% EtOH 5 min RT/70% EtOH 5 min RT/hydrate in MilliQ water
- Wash with 1xPBS for 5 min RT
- Air dry the slide.
- Incubate with 1xPBS for 2 min RT
- Incubate with cytosolic buffer 20 min at 4˚C
- Incubate with 1xPBS for 5 min RT
- Add 200 µl of Accessible Chromatin Labelling solution and incubate at 37°C for 2 h in a humidified chamber
- Add 20 µL of 0.5 M EDTA and 2 µg of RNase A are added to the labeling reaction and incubated at 37°C for 30 min
- Lysed cells on slide at room temperature with cell lysis buffer, 200 uL of ATL buffer (QIAGEN)

- Carefully transfer to 1.5 ml DNA Lo-bind E-tube and add 20 µl of proteinase K (200 units, NEB, P8107S) incubate it 65°C O/N
- Heat inactivate Proteinase K by incubating reaction tube at 95˚C for 2 min
- Add 2.5 units of Nt.CviPII, 50 µl of NEB buffer #2 to the reaction mix and adjust with MQ water upto 500 µl and incubate at 37 ˚C for 12-16 hrs
- Heat inactivate Nt.CviPII by incubating reaction tube at 65˚C for 15 min
- Add **15 µL** of Blocked Streptavidin magnetic beads, 1 mL of high salt buffer incubate by end to end rotation for 2 hr at 4 ˚C
- Wash beads with 1X High Salt buffer 4 times, 0.05% Triton (the accessible chromatin DNA remain bound to beads)
- Add 1 mL of 1X TE and incubate on end-over end rotator for 5 min at R/T
- Resuspend in 50 µl TE
- Use NEB Ultra II library prep reagents.

Note: if the digestion by Nt.CviPII is incomplete and higher than 1kb bands are visible in the library, the samples could be drop dialyzed before Nt.CviPII addition.
